# Synthesis and Antibacterial Activity Evaluation of Biphenyl and Dibenzofuran Derivatives as Potential Antimicrobial Agents against Antibiotic-Resistant Bacteria

**DOI:** 10.3390/cimb44090280

**Published:** 2022-09-07

**Authors:** Xing Wang, Hao-Yu Fu, Wei He, Yu-Ting Xiang, Ze-Cheng Yang, Yi Kuang, Sheng-Xiang Yang

**Affiliations:** Zhejiang Provincial Key Laboratory of Chemical Utilization of Forestry Biomass, College of Chemistry and Materials Engineering, Zhejiang A&F University, Hangzhou 311300, China

**Keywords:** biphenyls, aucuparin, phytoalexins, antibacterial activity, antibiotic-resistant bacteria

## Abstract

The escalating prevalence of antibiotic-resistant bacteria has led to a serious global public health problem; therefore, there is an urgent need for the development of structurally innovative antibacterial agents. In our study, a series of biphenyl and dibenzofuran derivatives were designed and synthesized by Suzuki-coupling and demethylation reactions in moderate to excellent yields (51–94% yield). Eleven compounds exhibited potent antibacterial activities against the prevalent antibiotic-resistant Gram-positive and Gram-negative pathogens, among which compounds 4′-(trifluoromethyl)-[1,1′-biphenyl]-3,4,5-triol (**6i**) and 5-(9*H*-carbazol-2-yl) benzene-1,2,3-triol (**6m**) showed the most potent inhibitory activities against methicillin-resistant *Staphylococcus aureus* and multidrug-resistant *Enterococcus faecalis* with MIC (minimum inhibitory concentration) values as low as 3.13 and 6.25 μg/mL, respectively. Compounds 3′,5′-dimethyl-[1,1′-biphenyl]-3,4,4′,5-tetraol (**6e**), 4′-fluoro-[1,1′-biphenyl]-3,4,5-triol (**6g**), and 4′-(trifluoromethyl)-[1,1′-biphenyl]-3,4,5-triol (**6i**) showed comparable inhibitory activities with ciprofloxacin to Gram-negative bacterium carbapenems-resistant *Acinetobacter baumannii*. Study of the structure–activity relationship indicated that a strong electron-withdrawing group on the A ring and hydroxyl groups on the B ring of biphenyls were beneficial to their antibacterial activities, and for benzo-heterocycles, *N*-heterocycle exhibited optimal antibacterial activity. These results can provide novel structures of antibacterial drugs chemically different from currently known antibiotics and broaden prospects for the development of effective antibiotics against antibiotic-resistant bacteria.

## 1. Introduction

Since penicillin was discovered in 1928, antibiotics have been commonly used to treat a variety of clinical diseases and have saved countless lives from fatal infections [1]. However, the overuse and abuse of antibiotics have led to a dramatic increase in bacterial resistances [2,3], which has posed a great threat to global public health and social development [4]. According to statistics from the World Health Organization (WHO), at least 700,000 people die each year due to drug-resistant diseases, including 230,000 people who die from multidrug-resistant tuberculosis. WHO also has issued a report calling for fast, coordinated, and ambitious action to avoid a devastating drug-resistance disaster. If the problem of antimicrobial resistances is not addressed effectively, drug-resistant diseases could cause ten million deaths a year by 2050 and economical damage as catastrophic as the 2008–2009 global financial crisis [5]. However, to date, no targeting therapeutic against resistant bacteria has received approval from the US Food and Drug Administration [6]. Therefore, this critical scenario has triggered global initiatives to innovate antimicrobial drugs and drug-resistant strains have attracted increasing attention. Prevalent multidrug-resistant bacteria belonging to the so-called ESKAPE (*Enterococcus faecium*, *Staphylococcus aureus*, *Klebsiella pneumoniae*, *Acinetobacter baumannii*, *Pseudomonas aeruginosa*, and *Enterobacter* species) panel, which has been defined as the top six critical bacterial pathogens by WHO, has been especially troublesome [7,8,9]. In the past few decades, the speed of new antibiotics development has failed to match that of drug-resistant bacteria production and many available antibiotics are failing against several critical pathogenic strains, which makes clinical treatment very difficult [10,11,12]. The vicious cycle between drug-resistance and new drug development, together with the fact that existing drugs still have difficulty in effectively controlling infection by new drug-resistant bacteria, make the development of new antidrug-resistant antibacterial drugs with a different mode of action particularly urgent in modern society.

There have been various antibiotic-resistant mechanisms reported to explain the causes of bacteria resistance, including the following eight factors: (i) increased efflux pumps lead to a decrease in intracellular antibiotics concentrations; (ii) acquisition and expression of drug resistant genes; (iii) modification of antimicrobial targets result in attenuated interactions with antibiotics; (iv) inactivating antibacterial drugs through the expression of drug-degrading enzymes; (v) decreased membrane permeability and altered metabolic state of bacteria; (vi) emergence of antibiotic-tolerant cells; (vii) biofilm formation; (viii) swarming [13]. These mechanisms ultimately lead to the lesser accumulation of antibiotics in bacterial cells and a stable, heritable ability of a microorganism to proliferate in the presence of high levels of an antibiotic, which in turn lower their therapeutic efficacy. Therefore, higher and repeated doses of antibiotics are required to defeat the bacteria. Currently, bactericidal mechanisms of the majority of clinically useful antibacterial drugs include three operations in the bacterial cell: cell-wall biosynthesis, protein synthesis, or enzymes involved in bacterial DNA replication [14].

**Figure 1:**
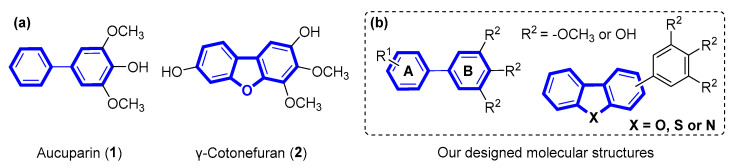
(**a**) Typical chemical structures of natural biphenyls and dibenzofurans phytoalexins and (**b**) our designed molecular structures.

To defuse the antibacterial resistance crisis, medicinal chemists are devoting their resources to innovate antibacterial drugs without pre-existing resistance against human bacterial pathogens by uncovering new molecular structures and utilizing unique modes of action and hybrid antibacterial drugs strategies [15,16,17,18,19,20,21,22,23,24]. Natural products can be regarded as a group of privileged structures that have been evolutionarily selected to interact with various biological targets; therefore, they are always an abundant source for antibacterial drug development [25,26,27]. Phytoalexins are a class of important low-molecular-weight secondary metabolites produced by plants as defense compounds to resist biotic and abiotic stresses [28,29]. As the chemical weapons of plants, they have shown biological activity against a wide range of pathogens and have potential as biological antimicrobial agents [30,31]. Biphenyls and dibenzofurans are two classes of important phytoalexins produced by Pyrinae, a species of the Rosaceous subtribe, when attacked by bacterial and fungal pathogens [28,32,33]. As shown in Figure 1a, aucuparin (1) and γ-cotonefuran (2) are the most widely distributed, and were first detected as defense compounds in the Pyrinae species in 1963 and 1984, respectively [34,35]. It has been reported in the literature that biphenyl derivatives exhibit a wide variety of biological properties, including anti-amoebic, antifungal, anti-infective, anti-hypercholesteremic, anti-hyperlipoproteinemic, antirheumatic, analgesic, anti-inflammatory, antithrombotic, uricosuric, and anti-arrhythmic properties [36,37]. For example, in our continuous efforts to develop new antimicrobials, we have found that some natural biphenyl-type phytoalexins, such as 3′,4′,5′-trimethoxy-[1,1′-biphenyl]-4-ol and 3,4,4′,5-tetramethoxy-1,1′-biphenyl, indeed showed significant activity against fungi [38]. However, research on their antibacterial activity against antibiotic-resistant bacteria is scarce. Lan-Ping Guo and colleagues were the first to report that dibenzofuran and biphenyl phytoalexins from a *Sorbus pohuashanensis* suspension cell exhibited potent antibacterial activity against drug-resistant bacteria [39]. These results provided our rationale in synthesizing novel biphenyl and dibenzofuran phytoalexin derivatives as potential antimicrobial agents against antibiotic-resistant bacteria.

As shown in Figure 1b, a diversity of biphenyl derivatives can be obtained by introducing different substituents on the A and B rings, dibenzofurans analogues can be produced via replacement of oxygen with a sulfur or nitrogen atom and substitution on the ring of corresponding benzo-heterocycles with aryls.

Therefore, in our study, a series of biphenyl and benzo-heterocycle phytoalexin derivatives were designed and synthesized, and their in vitro antibacterial activities against drug-resistant bacteria and structure–activity relationships were studied. From this study, we hope to find lead compounds with potent inhibitory activities as potential antidrug-resistant antibacterial drugs.

## 2. Materials and Methods

### 2.1. Chemistry

We recorded ^1^H NMR spectra with Varian Mercury 400/600 MHz spectrometers (Varian Associates, Inc., Palo Alto, CA, USA). Chemical shifts (*δ*) were reported in ppm, quoted relative to internal tetramethylsilane (internal standard, 0.0 ppm) with the coupling constants (*J*) given in Hz. We recorded ^13^C NMR spectra with the same spectrometer, operating at 100/150 MHz with complete proton decoupling (internal standard CDCl_3_: 77.0 ppm or DMSO-*d*_6_: 39.5 ppm). Splitting patterns were assigned s = singlet, d = doublet, t =triplet, dd = double doublet, dt = doublet of triplet, q = quartet, etc. High resolution mass spectrometry (HRMS) was performed on the Agilent Technologies 6530 Accurate-Mass Q-TOF mass spectrometer (Agilent Technologies, Santa Clara, CA, USA). Unless otherwise specified, all chemical reagents, biologics, and raw materials were purchased from Sigma-Aldrich (Shanghai, China) and could be used without special treatment. All reactions were monitored by thin layer chromatography (TLC) analysis on silica gel coated plates. Flash column chromatography was performed by using 200–300 mesh silica gel.

#### 2.1.1. General Procedure for the Synthesis of Compounds **5a**–**5n**

A mixture of bromobenzene (1 mmol) (Sigma-Aldrich, Shanghai, China), 3,4,5-trimethoxyphenylboronicacid (1.1 mmol) (Sigma-Aldrich, Shanghai, China), Pd(dppf)Cl_2_ (0.04 mmol) (Sigma-Aldrich, Shanghai, China), K_3_PO_4_ (3 mmol) (Sinopharm, Beijing, China), and H_2_O (5 mmol) in 1,4-dioxane (10 mL) (Sinopharm, Beijing, China)was stirred at 80 °C for 4 h under N_2_ atmosphere. The reaction progress was monitored by TLC. Upon completion of the reaction, the reaction mixture was poured into water (50 mL). The aqueous layer was extracted with CH_2_Cl_2_ (3 × 25 mL) (Sinopharm, Beijing, China), the combined organic phases were washed with saturated saline solution (3 × 100 mL), dried with anhydrous sodium sulfate, and concentrated under reduced pressure. The residue was purified by silica gel column chromatography on silica gel (Sinopharm, Beijing, China) with petroleum ether/ethyl acetate (Sinopharm, Beijing, China) (20:3, *v*/*v*) as eluent to afford the desired products **5a**–**5n** in 52–94% yields. Their ^1^H NMR and ^13^C NMR spectra can be found in the Supplementary Materials.

*3,4,5-Trimethoxy-1,1′-biphenyl* (**5a**): ^1^H NMR (400 MHz, CDCl_3_) δ 7.55 (d, *J* = 7.9 Hz, 2H), 7.43 (t, *J* = 7.5 Hz, 2H), 7.34 (t, *J* = 6.9 Hz, 1H), 6.78 (s, 2H), 3.92 (s, 6H), and 3.89 (s, 3H) ppm. ^13^C NMR (100 MHz, CDCl_3_) δ 153.37, 141.28, 137.50, 137.17, 128.66, 127.23, 127.01, 104.35, 60.88, and 56.10 ppm. HRMS: calculated for C_15_H_16_O_3_ [M + H]^+^: 245.1172, found: 245.1172.

*4′-Methyl-3,4,5-trimethoxy-1,1′-biphenyl* (**5b**): ^1^H NMR (400 MHz, CDCl_3_) δ 7.45 (d, *J* = 7.9 Hz, 2H), 7.24 (d, *J* = 7.2 Hz, 2H), 6.76 (s, 2H), 3.92 (s, 6H), 3.89 (s, 3H), and 2.39 (s, 3H) ppm. ^13^C NMR (100 MHz, CDCl_3_) δ 153.31, 138.38, 137.18, 137.13, 137.04, 129.38, 126.85, 104.04, 60.90, 56.06, and 21.03 ppm. HRMS: calculated for C_16_H_18_O_3_ [M + H]^+^: 259.1329, found: 259.1331.

*4′-Butyl-3,4,5-trimethoxy-1,1′-biphenyl* (**5c**): ^1^H NMR (600 MHz, CDCl_3_) δ 7.46 (d, *J* = 7.5 Hz, 2H), 7.24 (d, *J* = 7.0 Hz, 2H), 6.77 (s, 2H), 3.91 (s, 6H), 3.89 (s, 3H), 2.65 (t, *J* = 7.3 Hz, 2H), 1.71–1.54 (m, 2H), 1.39 (dd, *J* = 14.3, 7.1 Hz, 2H), and 0.94 (t, *J* = 7.1 Hz, 3H) ppm. ^13^C NMR (150 MHz, CDCl_3_) δ 153.30, 142.06, 138.60, 137.18, 128.72, 126.84, 104.12, 60.87, 56.05, 35.20, 33.60, 22.32, and 13.92 ppm. HRMS: calculated for C_19_H_24_O_3_ [M + H]^+^: 301.1798, found: 301.1811.

*3′-Methyl-3,4,4′,5-tetramethoxy-1,1′-biphenyl* (**5d**): ^1^H NMR (600 MHz, CDCl_3_) δ 7.42–7.27 (m, 2H), 6.87 (d, *J* = 8.0 Hz, 1H), 6.73 (s, 2H), 3.92 (s, 6H), 3.88 (s, 3H), 3.86 (s, 3H), and 2.29 (s, 3H) ppm. ^13^C NMR (150 MHz, CDCl_3_) δ 157.26, 153.25, 137.08, 136.89, 133.37, 129.28, 126.79, 125.22, 109.97, 103.90, 60.86, 56.05, 55.33, and 16.31 ppm. HRMS: calculated for C_17_H_20_O_4_ [M + H]^+^: 289.1434, found: 289.1456.

*3′,5′-Dimethyl-3,4,4′,5-tetramethoxy-1,1′-biphenyl* (**5e**): ^1^H NMR (400 MHz, CDCl_3_) δ 7.18 (s, 2H), 6.71 (s, 2H), 3.91 (s, 6H), 3.87 (s, 3H), 3.75 (s, 3H), and 2.34 (s, 6H) ppm. ^13^C NMR (100 MHz, CDCl_3_) δ 156.45, 153.22, 137.07, 137.00, 136.88, 131.08, 127.41, 104.06, 60.88, 59.73, 56.07, and 16.18 ppm. HRMS: calculated for C_18_H_22_O_4_ [M + H]^+^: 303.1591, found: 303.1588.

*3′,4′,5′-Trimethoxy-[1,1′-biphenyl]-2-ol* (**5f**): ^1^H NMR (400 MHz, CDCl_3_) δ 7.25 (dd, *J* = 12.9, 5.0 Hz, 2H), 6.98 (t, *J* = 8.5 Hz, 2H), 6.65 (s, 2H), 5.61 (s, 1H), 3.89 (s, 3H), and 3.87 (s, 6H) ppm. ^13^C NMR (100 MHz, CDCl_3_) δ 153.66, 152.42, 137.28, 132.56, 129.88, 129.10, 128.07, 120.58, 115.78, 105.84, 60.83, and 56.05 ppm. HRMS: calculated for C_15_H_16_O_4_ [M + H]^+^: 261.1121, found: 261.1175.

*4′-Fluoro-3,4,5-trimethoxy-1,1′-biphenyl* (**5g**): ^1^H NMR (600 MHz, CDCl_3_) δ 7.50 (s, 2H), 7.11 (t, *J* = 8.1 Hz, 2H), 6.72 (s, 2H), 3.92 (s, 6H), and 3.89 (s, 3H) ppm. ^13^C NMR (150 MHz, CDCl_3_) δ 163.09, 161.46, 153.36, 137.42, 137.35, 136.15, 128.55, 128.50, 115.53, 115.39, 104.18, 60.86, and 56.07 ppm. HRMS: calculated for C_15_H_15_FO_3_ [M + H]^+^: 263.1078, found: 263.1075.

*4′-Chloro-3,4,5-trimethoxy-1,1′-biphenyl* (**5h**): ^1^H NMR (600 MHz, CDCl_3_) δ 7.39 (d, *J* = 7.9 Hz, 2H), 7.30 (d, *J* = 7.8 Hz, 2H), 6.64 (s, 2H), 3.83 (s, 6H), and 3.81 (s, 3H) ppm. ^13^C NMR (150 MHz, CDCl_3_) δ 153.43, 139.67, 137.70, 135.86, 133.22, 128.77, 128.23, 104.15, 60.89, and 56.12 ppm. HRMS: calculated for C_15_H_15_ClO_3_ [M + H]^+^: 279.0782, found: 279.0779.

*3,4,5-Trimethoxy-4′-(trifluoromethyl)-1,1′-biphenyl* (**5i**): ^1^H NMR (400 MHz, CDCl_3_) δ 7.74–7.58 (m, 4H), 6.78 (s, 2H), 3.94 (s, 6H), and 3.91 (s, 3H) ppm. ^13^C NMR (100 MHz, CDCl_3_) δ 153.55, 144.77, 138.15, 135.63, 127.32, 125.64, 125.61, 125.57, 104.42, 60.94, and 56.16 ppm. HRMS: calculated for C_16_H_15_F_3_O_3_ [M + H]^+^: 313.1046, found: 313.1044.

*3-(3,4,5-Trimethoxyphenyl) dibenzo[b,d]furan* (**5j**): ^1^H NMR (400 MHz, CDCl_3_) δ 8.09 (s, 1H), 8.02 (d, *J* = 7.6 Hz, 1H), 7.62 (dt, *J* = 15.1, 8.3 Hz, 3H), 7.49 (t, *J* = 7.7 Hz, 1H), 7.37 (t, *J* = 7.5 Hz, 1H), 6.85 (s, 2H), 3.97 (s, 6H), and 3.92 (s, 3H) ppm. ^13^C NMR (100 MHz, CDCl_3_) δ 156.66, 155.69, 153.47, 137.37, 136.57, 127.38, 126.55, 124.66, 124.12, 122.80, 120.71, 119.07, 111.79, 111.71, 104.72, 60.99, and 56.24 ppm. HRMS: calculated for C_21_H_18_O_4_ [M + H]^+^: 335.1278, found: 335.1301.

*3-(3,4,5-Trimethoxyphenyl) dibenzo[b,d]thiophene* (**5k**): ^1^H NMR (600 MHz, CDCl_3_) δ 8.27 (s, 1H), 8.21 (s, 1H), 7.86 (dd, *J* = 11.2, 5.4 Hz, 2H), 7.63 (d, *J* = 7.4 Hz, 1H), 7.46 (s, 2H), 6.86 (s, 2H), 3.95 (s, 6H), and 3.92 (s, 3H) ppm. ^13^C NMR (150 MHz, CDCl_3_) δ 153.44, 139.82, 138.38, 137.96, 137.50, 137.15, 135.91, 135.31, 126.83, 126.06, 124.34, 122.87, 121.56, 119.80, 104.57, 60.92, and 56.17 ppm. HRMS: calculated for C_21_H_18_O_3_S [M + H]^+^: 351.1049, found: 351.1068.

*3-(3,4,5-Trimethoxyphenyl)-9H-carbazole* (**5l**): ^1^H NMR (600 MHz, CDCl_3_) δ 8.32 (s, 1H), 8.23 (s, 1H), 8.13 (d, *J* = 7.1 Hz, 1H), 7.60 (d, *J* = 7.2 Hz, 1H), 7.46–7.35 (m, 3H), 7.24 (dd, *J* = 14.3, 7.6 Hz, 1H), 6.88 (s, 2H), 3.95 (s, 6H), and 3.93 (s, 3H) ppm. ^13^C NMR (150 MHz, CDCl_3_) δ 153.31, 139.97, 138.95, 138.30, 136.80, 132.87, 125.98, 125.24, 123.60, 123.14, 120.26, 119.38, 118.58, 110.76, 104.50, 60.93, and 56.12 ppm. HRMS: calculated for C_21_H_19_NO_3_ [M + H]^+^: 334.1438, found: 334.1442.

*2-(3,4,5-Trimethoxyphenyl)-9H-carbazole* (**5m**): ^1^H NMR (600 MHz, CDCl_3_) δ 8.23 (s, 1H), 7.99 (t, *J* = 8.1 Hz, 2H), 7.43 (s, 1H), 7.32 (d, *J* = 16.2 Hz, 3H), 7.14 (d, *J* = 5.2 Hz, 1H), 6.78 (s, 2H), 3.84 (s, 3H), and 3.83 (s, 6H) ppm. ^13^C NMR (150 MHz, CDCl_3_) δ 153.30, 139.95, 139.20, 138.08, 137.23, 125.80, 122.93, 122.51, 120.37, 120.24, 119.44, 118.89, 110.63, 109.04, 104.68, 104.49, 60.94, and 56.11 ppm. HRMS: calculated for C_21_H_19_NO_3_ [M + H]^+^: 334.1438, found: 334.1448.

*1-(3,4,5-Trimethoxyphenyl)-9H-carbazole* (**5n**): ^1^H NMR (600 MHz, CDCl_3_) δ 8.47 (s, 1H), 8.10 (d, *J* = 7.5 Hz, 1H), 8.06 (d, *J* = 7.4 Hz, 1H), 7.48–7.34 (m, 3H), 7.29 (t, *J* = 7.4 Hz, 1H), 7.23 (dd, *J* = 6.6, 4.4 Hz, 1H), 6.85 (s, 2H), 3.93 (s, 3H), and 3.88 (s, 6H) ppm. ^13^C NMR (150 MHz, CDCl_3_) δ 153.67, 139.45, 137.32, 137.24, 134.70, 125.90, 125.39, 125.17, 123.60, 123.42, 120.39, 119.64, 119.46, 119.39, 110.74, 105.46, 60.90, and 56.18 ppm. HRMS: calculated for C_21_H_19_NO_3_ [M + H]^+^: 334.1438, found: 334.1422.

#### 2.1.2. General Procedure for the Synthesis of Compounds **6a**–**6n**

To a stirred solution of Compound 5 (0.5 mmol) in anhydrous CH_2_Cl_2_ (10 mL), we added BBr_3_ (Sigma-Aldrich, Shanghai, China) (1 mol/L in CH_2_Cl_2_, 1.8 equiv.) in portions at 0 °C under nitrogen atmosphere. The reaction mixture was allowed to warm to room temperature and stirred overnight. The reaction progress was monitored by TLC. Upon completion of the reaction, the mixture was poured into water (50 mL). The aqueous layer was extracted with EtOAc (Sinopharm, Beijing, China) (3 × 25 mL), the combined organic phases were washed with saturated saline solution (100 mL × 3), dried with anhydrous sodium sulfate, and concentrated under reduced pressure. The residue was purified by silica gel column chromatography using 75–90% EtOAc in petroleum ether as eluent to afford the desired products **6a**–**6n** in 51–84% yields. Their ^1^H NMR and ^13^C NMR spectra can be found in the Supplementary Materials.

*[1,1′-Biphenyl]-3,4,5-triol* (**6a**): ^1^H NMR (400 MHz, DMSO-*d*_6_) δ 8.99 (s, 2H), 8.29 (s, 1H), 7.46 (d, *J* = 7.3 Hz, 2H), 7.38 (t, *J* = 7.6 Hz, 2H), 7.24 (t, *J* = 7.3 Hz, 1H), and 6.57 (s, 2H) ppm. ^13^C NMR (100 MHz, DMSO-*d*_6_) δ 146.52, 146.52, 140.78, 140.78, 133.06, 133.06, 130.79, 130.79, 128.80, 128.80, 126.41, 126.41, 125.97, 125.97, 105.58, and 105.58 ppm. HRMS: calculated for C_12_H_10_O_3_ [M + H]^+^: 201.0557, found: 201.0551.

*4′-Methyl-[1,1′-biphenyl]-3,4,5-triol* (**6b**): ^1^H NMR (400 MHz, DMSO-*d*_6_) δ 8.96 (s, 2H), 8.24 (s, 1H), 7.36 (d, *J* = 7.9 Hz, 1H), 7.18 (d, *J* = 7.8 Hz, 1H), 6.56 (s, 1H), and 2.29 (s, 3H) ppm. ^13^C NMR (100 MHz, DMSO-*d*_6_) δ 146.50, 137.93, 135.50, 132.80, 130.81, 129.39, 125.82, 105.39, and 20.65 ppm. HRMS: calculated for C_13_H_12_O_3_ [M-H]^−^: 215.0714, found: 215.0707.

*4′-Butyl-[1,1′-biphenyl]-3,4,5-triol* (**6c**): ^1^H NMR (400 MHz, DMSO-*d*_6_) δ 8.95 (s, 2H), 8.22 (s, 1H), 7.37 (d, *J* = 8.0 Hz, 2H), 7.18 (d, *J* = 8.0 Hz, 2H), 6.56 (s, 2H), 2.54 (dd, *J* = 17.9, 10.3 Hz, 2H), 1.54 (dt, *J* = 15.1, 7.6 Hz, 2H), 1.29 (dq, *J* = 14.6, 7.4 Hz, 2H), and 0.88 (t, *J* = 7.3 Hz, 3H) ppm. ^13^C NMR (100 MHz, DMSO-*d*_6_) δ 146.48, 140.43, 138.16, 132.80, 130.82, 128.69, 125.80, 105.39, 34.45, 33.19, 21.82, and 13.85 ppm. HRMS: calculated for C_16_H_18_O_3_ [M + H]^+^: 259.1329, found: 259.1338.

*3′-Methyl-[1,1′-biphenyl]-3,4,4′,5-tetraol* (**6d**): ^1^H NMR (400 MHz, DMSO-*d*_6_) δ 9.26 (s, 1H), 8.86 (s, 2H), 8.11 (s, 1H), 7.18 (s, 1H), 7.11 (dd, *J* = 8.2, 1.7 Hz, 1H), 6.80 (d, *J* = 8.3 Hz, 1H), 6.49 (s, 2H), and 2.17 (s, 3H) ppm. ^13^C NMR (100 MHz, DMSO-*d*_6_) δ 154.44, 146.46, 132.10, 131.69, 131.39, 128.38, 124.29, 124.07, 115.00, 105.13, and 16.32 ppm. HRMS: calculated for C_13_H_12_O_4_ [M + H]^+^: 233.0808, found: 233.0823.

*3′,5′-Dimethyl-[1,1′-biphenyl]-3,4,4′,5-tetraol* (**6e**): ^1^H NMR (400 MHz, DMSO-*d*_6_) δ 8.83 (s, 2H), 8.19 (s, 1H), 8.10 (s, 1H), 7.02 (s, 2H), 6.47 (s, 2H), and 2.20 (s, 6H) ppm. ^13^C NMR (100 MHz, DMSO-*d*_6_) δ 152.19, 146.37, 132.05, 131.84, 131.30, 125.89, 124.41, 105.08, and 16.90 ppm. HRMS: calculated for C_14_H_14_O_4_ [M + H]^+^: 247.0965, found: 247.0953.

*[1,1′-Biphenyl]-2,3′,4′,5′-tetraol* (**6f**): ^1^H NMR (400 MHz, DMSO-*d*_6_) δ 9.24 (s, 1H), 7.12 (d, *J* = 7.4 Hz, 1H), 7.06 (t, *J* = 7.7 Hz, 1H), 6.86 (d, *J* = 7.8 Hz, 1H), 6.80 (t, *J* = 7.5 Hz, 1H), and 6.48 (s, 2H) ppm. ^13^C NMR (100 MHz, DMSO-*d*_6_) δ 154.10, 145.47, 132.07, 129.88, 128.79, 128.27, 127.39, 119.22, 115.93, and 108.17 ppm. HRMS: calculated for C_12_H_10_O_4_ [M-H]^−^: 217.0506, found: 217.0501.

*4′-Fluoro-[1,1′-biphenyl]-3,4,5-triol* (**6g**): ^1^H NMR (400 MHz, DMSO-*d*_6_) δ 9.01 (s, 2H), 8.31 (s, 1H), 7.49 (dd, *J* = 8.5, 5.6 Hz, 2H), 7.19 (t, *J* = 8.8 Hz, 2H), and 6.54 (s, 2H) ppm. ^13^C NMR (100 MHz, DMSO-*d*_6_) δ 162.47, 160.06, 146.57, 137.32, 137.29, 133.06, 129.88, 128.81, 127.84, 127.76, 125.99, 115.62, 115.41, and 105.64 ppm. HRMS: calculated for C_12_H_9_FO_3_ [M+2H]^2+^: 111.0341, found: 111.0377.

*4′-Chloro-[1,1′-biphenyl]-3,4,5-triol* (**6h**): ^1^H NMR (400 MHz, DMSO-*d*_6_) δ 9.10 (s, 2H), 8.47 (s, 1H), 7.96 (d, *J* = 7.8 Hz, 2H), 7.60 (d, *J* = 7.9 Hz, 2H), and 6.66 (s, 2H) ppm. ^13^C NMR (100 MHz, DMSO-*d*_6_) δ 167.38, 146.64, 144.97, 134.03, 130.02, 129.47, 128.54, 125.93, and 105.91 ppm. HRMS: calculated for C_12_H_9_ClO_3_ [M-H]^−^: 235.0167, found: 235.0158.

*4′-(Trifluoromethyl)-[1,1′-biphenyl]-3,4,5-triol* (**6i**): ^1^H NMR (400 MHz, DMSO-*d*_6_) δ 9.12 (s, 2H), 8.48 (s, 1H), 7.70 (q, *J* = 8.3 Hz, 4H), and 6.65 (s, 2H) ppm. ^13^C NMR (100 MHz, DMSO-*d*_6_) δ 146.66, 144.72, 134.09, 128.98, 126.53, 125.68, 125.64, 123.21, and 105.91 ppm. HRMS: calculated for C_13_H_9_F_3_O_3_ [M-H]^−^: 269.0431, found: 269.0420.

*5-(Dibenzo[b,d]furan-3-yl)benzene-1,2,3-triol* (**6j**): ^1^H NMR (400 MHz, DMSO-*d*_6_) δ 9.00 (s, 2H), 8.29 (s, 1H), 8.25 (s, 1H), 8.22 (d, *J* = 7.6 Hz, 1H), 7.69 (dd, *J* = 7.9, 4.2 Hz, 2H), 7.62 (d, *J* = 8.5 Hz, 1H), 7.51 (t, *J* = 7.5 Hz, 1H), 7.40 (t, *J* = 7.4 Hz, 1H), and 6.70 (s, 2H) ppm. ^13^C NMR (100 MHz, DMSO-*d*_6_) δ 155.93, 154.52, 146.58, 136.45, 132.87, 130.95, 127.64, 125.95, 124.07, 123.76, 123.10, 121.40, 118.39, 111.71, 111.69, and 106.06 ppm. HRMS: calculated for C_18_H_12_O_4_ [M-H]^−^: 291.0663, found: 291.0664.

*5-(Dibenzo[b,d]thiophen-3-yl)benzene-1,2,3-triol* (**6k**): ^1^H NMR (400 MHz, DMSO-*d*_6_) δ 9.05 (s, 2H), 8.46 (s, 2H), 8.37 (s, 1H), 8.12–7.89 (m, 2H), 7.64 (d, *J* = 7.4 Hz, 1H), 7.51 (dd, *J* = 5.7, 3.1 Hz, 2H), and 6.76 (s, 2H) ppm. ^13^C NMR (100 MHz, DMSO-*d*_6_) δ 146.61, 139.08, 137.92, 136.63, 135.65, 135.17, 133.14, 130.72, 127.13, 125.59, 124.77, 123.21, 123.09, 122.25, 119.16, and 106.09 ppm. HRMS: calculated for C_18_H_12_O_3_S [M-H]^−^: 307.0434, found: 307.0434.

*5-(9H-carbazol-3-yl) benzene-1,2,3-triol* (**6l**): ^1^H NMR (400 MHz, DMSO-*d*_6_) δ 11.22 (s, 1H), 8.98 (s, 2H), 8.24 (s, 1H), 8.09 (dd, *J* = 7.8, 3.5 Hz, 2H), 7.53 (s, 1H), 7.48 (d, *J* = 8.1 Hz, 1H), 7.37 (t, *J* = 7.6 Hz, 1H), 7.30 (d, *J* = 8.2 Hz, 1H), 7.15 (t, *J* = 7.4 Hz, 1H), and 6.68 (s, 2H) ppm. ^13^C NMR (100 MHz, DMSO-*d*_6_) δ 146.46, 140.41, 140.09, 138.50, 132.78, 131.72, 125.30, 122.33, 121.01, 120.35, 120.02, 118.58, 117.30, 110.84, 107.92, 105.79, and 66.35 ppm. HRMS: calculated for C_18_H_13_NO_3_ [M-H]^−^: 290.0823, found: 290.0809.

*5-(9H-carbazol-2-yl) benzene-1,2,3-triol* (**6m**): ^1^H NMR (400 MHz, DMSO-*d*_6_) δ 11.24 (s, 1H), 8.96 (s, 2H), 8.32–8.04 (m, 3H), 7.52 (dt, *J* = 15.9, 8.1 Hz, 3H), 7.38 (t, *J* = 7.4 Hz, 1H), 7.16 (t, *J* = 7.3 Hz, 1H), and 6.70 (s, 2H) ppm. ^13^C NMR (100 MHz, DMSO-*d*_6_) δ 146.55, 140.25, 138.79, 132.25, 132.22, 131.88, 125.66, 124.35, 122.96, 122.64, 120.37, 118.59, 117.39, 111.16, 111.07, and 105.80 ppm. HRMS: calculated for C_18_H_13_NO_3_ [M-H]^−^: 290.0823, found: 290.0816.

*5-(9H-carbazol-1-yl) benzene-1,2,3-triol* (**6n**): ^1^H NMR (400 MHz, DMSO-*d*_6_) δ 11.25 (s, 1H), 9.04 (s, 2H), 8.31 (s, 1H), 8.08 (t, *J* = 7.1 Hz, 2H), 7.55 (s, 1H), 7.49 (d, *J* = 8.0 Hz, 1H), 7.37 (t, *J* = 7.6 Hz, 1H), 7.31 (d, *J* = 8.1 Hz, 1H), 7.14 (t, *J* = 7.4 Hz, 1H), and 6.70 (s, 2H) ppm. ^13^C NMR (100 MHz, DMSO) δ 146.54, 140.48, 140.15, 138.57, 132.86, 131.82, 125.37, 122.39, 121.08, 120.41, 120.08, 118.65, 117.38, 110.90, 108.00, and 105.88 ppm. HRMS: calculated for C_18_H_13_NO_3_ [M-H]^−^: 290.0823, found: 290.0823.

### 2.2. Antibacterial Activity Assays

MIC Testing. All synthesized target compounds were evaluated for their antibacterial activities in vitro against four Gram-positive (methicillin-resistant *Staphylococcus aureus* (MRSA), multidrug-resistant *Staphylococcus epidermidis* (MRSE), multidrug-resistant *Enterococcus faecium* (MREF), and multidrug-resistant *Enterococcus faecalis* (MREf)) and four Gram-negative (carbapenems-resistant *Pseudomonas aeruginosa* (CRPA)*,* carbapenems-resistant *Acinetobacter baumannii* (CRAB), carbapenems-resistant *Klebsiella pneumoniae* (CRKP), and carbapenems-resistant *Escherichia coli* (CREC)) bacteria. They were grown in a sterile liquid LB medium (Sangon Biotech Co., Ltd. Shanghai, China) (yeast extract 5 g/L, peptone 10 g/L, NaCl 10 g/L, pH = 7.4) overnight at 37 °C, and the diluted bacterial suspension (10^6^ CFU per milliliter) was ready for detection. The minimum inhibitory concentration (MIC) of samples and positive control were determined in sterile 96-well microplates by the modified broth dilution test method [40]. All wells were filled with 90 μL of bacterial suspension containing 10^6^ CFU per milliliter. Test samples (10 μL) with their different concentrations were added into each well. Samples that were difficult to dissolve were sonicated. Media containing 1% DMSO and ciprofloxacin were used as negative and positive controls, respectively. The final concentrations of ciprofloxacin and test compounds were 100, 50, 25, 12.5, 6.25, 3.125, 1.5625, and 0.78125 μg/mL in medium. Finally, plates were incubated at 37 °C for 12–36 h, and the results were observed by the naked eye. The minimum inhibitory concentration (MIC) was defined as the lowest test concentration that inhibited the growth of the test bacteria. The data was acquired from three independent assays performed in triplicate. All tested bacterial strains for biological studies were gifts from The Third Military Medical University (Chongqing, China).

## 3. Results

### 3.1. Design and Synthesis of Biphenyl and Benzo-Heterocycle Phytoalexin Derivatives

**Scheme 1:**
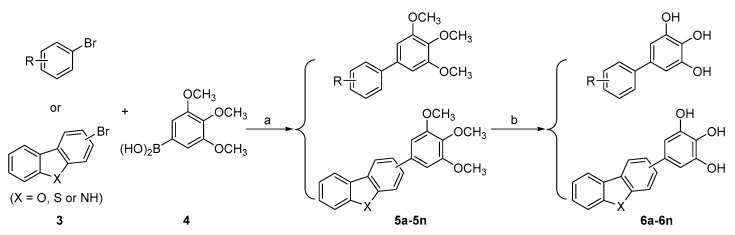
Synthesis routes of target Compounds **5** and **6**. Reagents and conditions: (**a**) ArBr (1 mmol), 3,4,5-trimethoxyphenylboronicacid (1.1 mmol), Pd(dppf)Cl_2_ (0.04 mmol), K_3_PO_4_ (3 mmol), H_2_O (5 mmol), and 1,4-dioxane (10 mL), under N_2_, at 80 °C, over 4 h; (**b**) 3,4,5-Trimethoxybiphenyl 5 (1 mmol), anhydrous CH_2_Cl_2_ (10 mL), and BBr_3_ (1 mol/L in CH_2_Cl_2_, 1.8 mL), under N_2_, from 0 °C to room temperature, overnight.

**Scheme 2:**
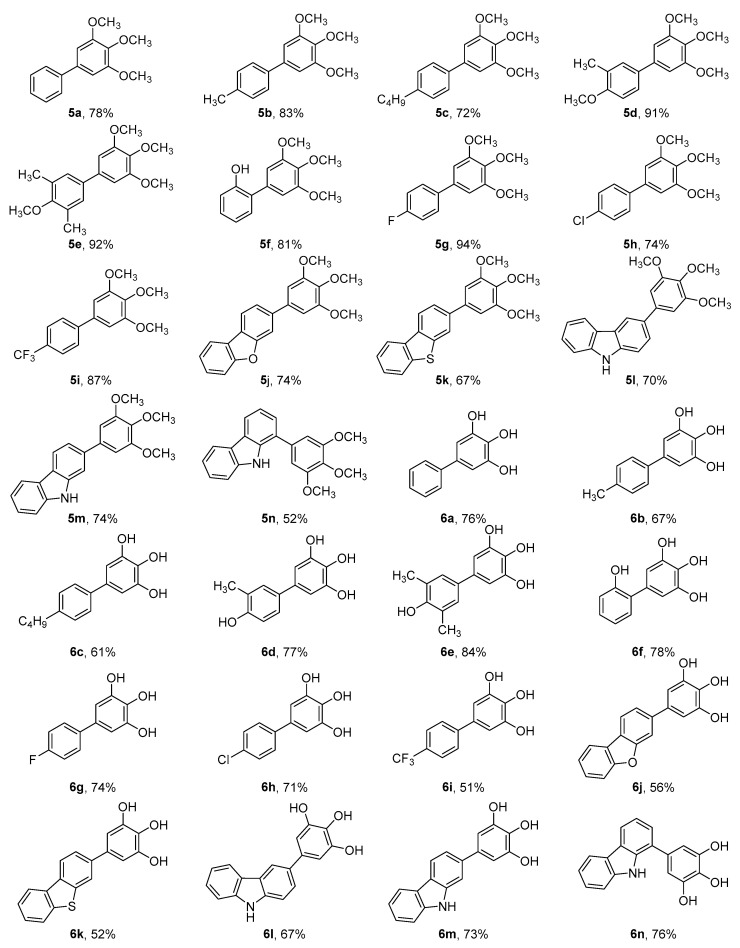
List of target compounds with corresponding yields.

As shown in Scheme 1, two series of biphenyl and benzo-heterocycle phytoalexin derivatives could be conveniently synthesized. First, the palladium-catalyzed Suzuki–Miyaura coupling reaction between aryl bromides (3) and 3,4,5-trimethoxyphenylboronic acid (4) in the presence of the weak base, K_3_PO_4_, gave the target Compounds **5a**–**5n** in 52–94% yields [41,42]. Then, the demethylation of Compounds **5a**–**5n** with BBr_3_, under nitrogen atmosphere, produced polyhydroxy Compounds **6a**–**6n** in 51–84% yields. The structures of these target compounds were confirmed by ^1^H NMR, ^13^C NMR, and HRMS characterization analyses and their structures are shown in Scheme 2.

### 3.2. Antibacterial Activities

The emergence of novel antibiotic-resistant bacterial strains is considered one of the biggest public health problems of the 21st century. Hundreds of thousands of people die every year from infection by antibiotic-resistant bacteria [43], leading to an estimated cost of at least EUR 1.5 billion/year, according to the European Centre for Disease Prevention and Control/European Medicines Agency (ECDC/EMEA) [44]. This group of bacteria includes *Enterococcus faecium*, *Staphylococcus aureus*, *Klebsiella pneumoniae*, *Acinetobacter baumannii*, *Pseudomonas aeruginosa*, and *Enterobacter* species, which are clinically important drug-resistant bacterial strains and characterized by high drug-resistance mechanisms [8]. We evaluated the in vitro antibacterial activities of 28 as-synthesized target compounds against the eight clinically important drug-resistant bacterial strains using a MIC broth dilution test method, as reported in the literature [40], which is a robust method for semi-quantifying antimicrobial activities of a large number of potential candidates. As shown in Table 1, the series of Compounds **5a**–**5n** exhibited no inhibition of the drug-resistant bacteria, with MIC values greater than 100 μg/mL. To our delight, their demethylation products, **6a**–**6n**, with polyhydroxyls showed moderate to significant inhibitory effects on Gram-positive bacteria and a few compounds also showed good inhibitory effects on Gram-negative bacteria, especially for the CRAB strain.

According to the MIC values of the preliminary screening results, 11 out of the 28 synthesized target compounds displayed effective antibacterial activities against Gram-positive bacteria, particularly compounds **6i** and **6m**, which exhibited the most potent inhibitory activities against MRSA and MREf with MIC values of 3.13 (ciprofloxacin: 1.56 μg/mL) and 6.25 μg/mL (ciprofloxacin: <0.78 μg/mL), respectively. Compounds **6a**, **6b**, **6d**, **6e**, **6f**, **6g**, and **6i** could effectively inhibit the MRSE with MIC values from 12.5 to 25 μg/mL (ciprofloxacin: 25 μg/mL). Encouragingly, Compounds **6a**, **6b**, **6d**, **6e**, **6g**, and **6i** showed good inhibitory activities not only against Gram-positive bacteria, but also against the Gram-negative bacterium, CRAB, with the same MIC value as ciprofloxacin (50 μg/mL). These results may provide new molecular skeletons for the discovery of drugs against resistant bacteria.

## 4. Discussion

Resistant strains of bacteria have spread all over the world, mostly in hospitals where so-called nosocomial polyresistant, or multidrug-resistant (MDR), strains are present. Due to the emergence of antibiotic resistance, bacterially mediated infectious diseases have become a major health concern over the word. The current situation of antibiotic resistance development underscores a pressing need to develop a novel class of antibiotics that has high efficacy in killing pathogens with acquired resistance to supersede existing antibiotics. Dibenzofurans and biphenyls are two classes of typical natural phytoalexins that are produced in plants after infection by fungal and bacterial pathogens, and some of them have shown significant antibacterial activities against antibiotic-resistant bacteria [39]. However, accessing these phytoalexins is generally done by isolating from plant tissues or suspension cells, making them difficult to use for new drug development due to their limited quantities and tedious extraction process.

In this work, two series of biphenyl and benzo-heterocycle phytoalexin derivatives are obtained by chemical synthesis using Suzuki–Miyaura coupling [44] and demethylation reactions [45], which can provide more compounds with rich structural types for activity screening. Aryl bromides with either electron-withdrawing or electron-donating substitutions work well in the reaction and provide the product in good yields. For example, 3,5-dimethyl-4-methoxylphenyl bromide and 4-fluorophenyl bromide gave corresponding products in 92 and 91% yields, respectively. The yields of demethylation products were slightly decreased. Antibacterial activity assays indicated that most of Compound **6** exhibited significant antibacterial activities against antibiotic-resistant Gram-positive strains but no obvious effects on Gram-negative strains, which demonstrated that these as-synthesized biphenyl and dibenzofuran derivatives have certain selectivity. The MIC values to MRSA of Compounds **6i** and **6m** were, respectively, 6.25 and 3,13 μg/mL, which are comparable to values reported in the literature [39,46]. On the other hand, the MIC value to MREF of Compound **6l** was 6.25 μg/mL, which indicates greater inhibitory activity than that of 4-methoxy-1-(methylthio)dibenzo[*b,d*]furan-2,3-diol (MIC = 12.5 μg/mL), reported by Gao [39].

The study of structure–activity relationships of as-synthesized compounds indicated that the hydroxyl groups on the B ring of biphenyl phytoalexin derivatives play a key role in exerting antibacterial activities, because antibacterial activities were completely lost when the hydroxyl groups were replaced with methoxyl groups (**5a**–**5n**). However, the hydroxyl group on the A ring did not benefit their antibacterial activities (**6f** vs. **6a**). For the A ring of the biphenyl series, the compound with a strong electron-withdrawing trifluoromethyl group (**6i**) instead of a methyl group (**6b**) was more efficient in inhibiting Gram-positive bacteria, which may be due to the hydrogen bond interaction between Compound **6i** and the potential target protein. When the methyl group was replaced with a longer *n*-butyl carbon chain (**6c**) or a chloro group (**6h**), this led to a complete loss of antibacterial activity. Increasing the number of substituents on the A ring was seemingly not beneficial to inhibitory activity, because antibacterial activities were significantly decreased (**6d,e** vs. **6b**). For the benzo-heterocycle series, the antibacterial activities of nitrogen heterocyclic carbazoles (**6l–6n**) were superior to oxygen or sulfur heterocyclic dibenzofuran (**6j**) and dibenzothiophene (**6k**), indicating that the nitrogen atom makes a significant contribution to = antibacterial activity. The position of the trihydroxyphenyl group on the carbazole ring had some influence on inhibitory effect; for example, the antibacterial activity of 2-trihydroxyphenyl group substituted carbazole was more potent than 1- and 3-position substituted Compounds (**6m** vs. **6l**,**n**).

The predicted results of the structure–activity relationship studies illustrated that hydroxyl groups and the nitrogen atom, which can form hydrogen bond interactions with target proteins in the typical manner of drug mechanisms, are essential for bactericidal activities. The results of the selectivity and structure–activity relationship studies of the synthesized compounds suggest that the bacteriostatic mechanism of these compounds may be the interaction with the key proteins of bacteria.

Given the convenience of synthesizing biphenyl and benzo-heterocycle phytoalexin derivatives and the possibility of structural diversification by changing the type, position, and number of substituents, there is considerable room for future improvement and optimization. In a word, these biphenyl and benzo-heterocycle phytoalexin derivatives have potential to be used in the development of new antimicrobial agents.

## 5. Conclusions

In conclusion, we have designed and synthesized a series of biphenyl and dibenzofuran derivatives and evaluated their in vitro antibacterial activities against drug-resistant bacteria. The results demonstrated that most of the polyhydric compounds exhibited moderate to significant inhibitory activities against prevalent drug-resistant Gram-positive and Gram-negative bacteria. Among these compounds, 11 out of the 28 synthesized target compounds displayed effective antibacterial activities against Gram-positive bacteria, especially Compounds **6i** and **6m**, and their MIC values could be as low as 3.13 μg/mL. Compounds **6a**, **6b**, **6d**, **6e**, **6f**, **6g**, and **6a** could effectively inhibit the multidrug-resistant *Staphylococcus epidermidis* with MIC values from 12.5 to 25 μg/mL, and simultaneously possess strong inhibitory effects against Gram-negative bacterium carbapenems-resistant *Acinetobacter baumannii*, except for 6f. The study of structure–activity relationships revealed that a strong electron-withdrawing group on the A ring and hydroxyl groups on the B ring of biphenyls are beneficial to their antibacterial activities, and for benzo-heterocycles, a nitrogen atom contributed more to antibacterial activity than oxygen or sulfur atoms. These results can provide new molecular structures for the discovery of effective antibiotics against drug-resistant bacteria.

## Figures and Tables

**Table 1:** In vitro antibacterial activities of 28 target compounds *^a^*.

Compounds	MIC *^b^* (μg/mL)							
	MRSA	MRSE *^c^*	MREF *^c^*	MREf *^c^*	CRPA	CRAB	CRKP	CREC
**5a**–**5n**	>100	>100	>100	>100	>100	>100	>100	>100
**6a**	12.5	12.5	12.5	50	50	50	100	100
**6b**	12.5	25	25	12.5	100	50	100	100
**6c**	>100	>100	>100	>100	>100	>100	>100	>100
**6d**	25	12.5	50	25	100	50	50	100
**6e**	25	25	50	25	100	50	100	100
**6f**	25	25	50	12.5	>100	100	>100	>100
**6g**	25	25	25	12.5	100	50	100	100
**6h**	>100	>100	>100	>100	>100	>100	>100	>100
**6i**	6.25	25	12.5	6.25	>100	50	100	100
**6j**	>100	>100	>100	>100	>100	>100	>100	>100
**6k**	>100	>100	>100	6.25	>100	>100	>100	>100
**6l**	6.25	>100	6.25	>100	>100	>100	>100	>100
**6m**	3.13	>100	>100	6.25	>100	>100	>100	>100
**6n**	12.5	100	12.5	12.5	>100	>100	>100	>100
Ciprofloxacin	1.56	25	3.13	<0.78	<0.78	50	>100	50

*^a^* Results of three independent experiments performed in triplicate. *^b^* Minimum inhibitory concentrations (MICs) were determined as the lowest concentration of the target compounds that inhibited bacterial growth with a modified broth dilution test method. *^c^* Multidrug-resistant (penicillin, tetracycline, and erythromycin) bacterial strains.

## Data Availability

All data are contained within the article or Supplementary Material.

## Supplementary Materials

The following supporting information can be downloaded at: https://www.mdpi.com/article/10.3390/cimb44090280/s1, copies of the ^1^H NMR and ^13^C NMR spectra for all target Compounds **5a**–**5n** and **6a**–**6n**.
