# Supplementary material for: Synthesis and Antibacterial Activity Evaluation of Biphenyl and Dibenzofuran Derivatives as Potential Antimicrobial Agents against Antibiotic-Resistant Bacteria
Source: Curr Issues Mol Biol. 2022 Sep 7;44(9):4087–99. doi: 10.3390/cimb44090280 (PMC9497828; doi:10.3390/cimb44090280)
Supplement: Supplementary file 1 [file cimb-44-00280-s001.zip › cimb-1879580-supplementary.pdf]

Supplementary Materials

Synthesis and Antibacterial Activity Evaluation of Aucuparin Phytoalexin Derivatives as Potential Antimicrobial Agents Against Antibiotic-resistant Bacteria

Xing Wang [1,†], Hao-Yu Fu [1,†], Wei He [1], Yu-Ting Xiang [1], Ze-Cheng Yang [1], Yi Kuang [1*] and Sheng-Xiang Yang [1,*]

[1] *Zhejiang Provincial Key Laboratory of Chemical Utilization of Forestry Biomass, Zhejiang A＆F University, Hangzhou 311300, China*

* Correspondence: kuangyi@zafu.edu.cn (Y.K.); shengxiangyang2000@zafu.edu.cn (S.-X.Y.)

† These authors have contributed equally to this work and share first authorship

Table of Content:

Copies of the ^{1}H NMR and ^{13}C NMR spectra of all target compounds

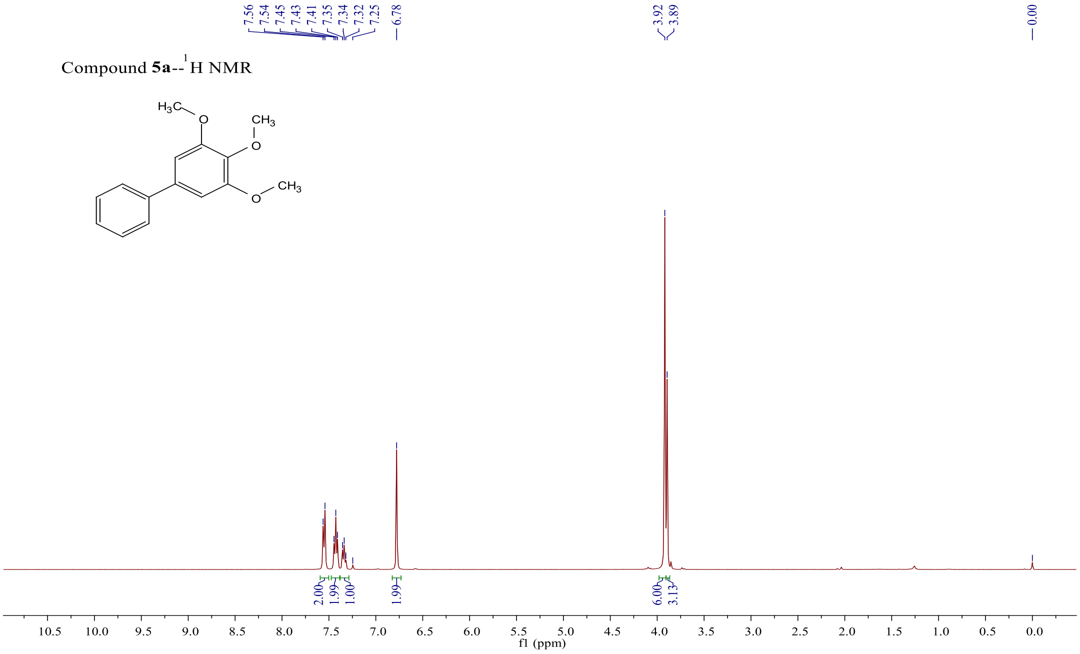

^{1}H NMR spectrum of compound **5a** (20 mg in 0.6 mL $CDCl_3$, 25 °C, Scan times ns = 32)

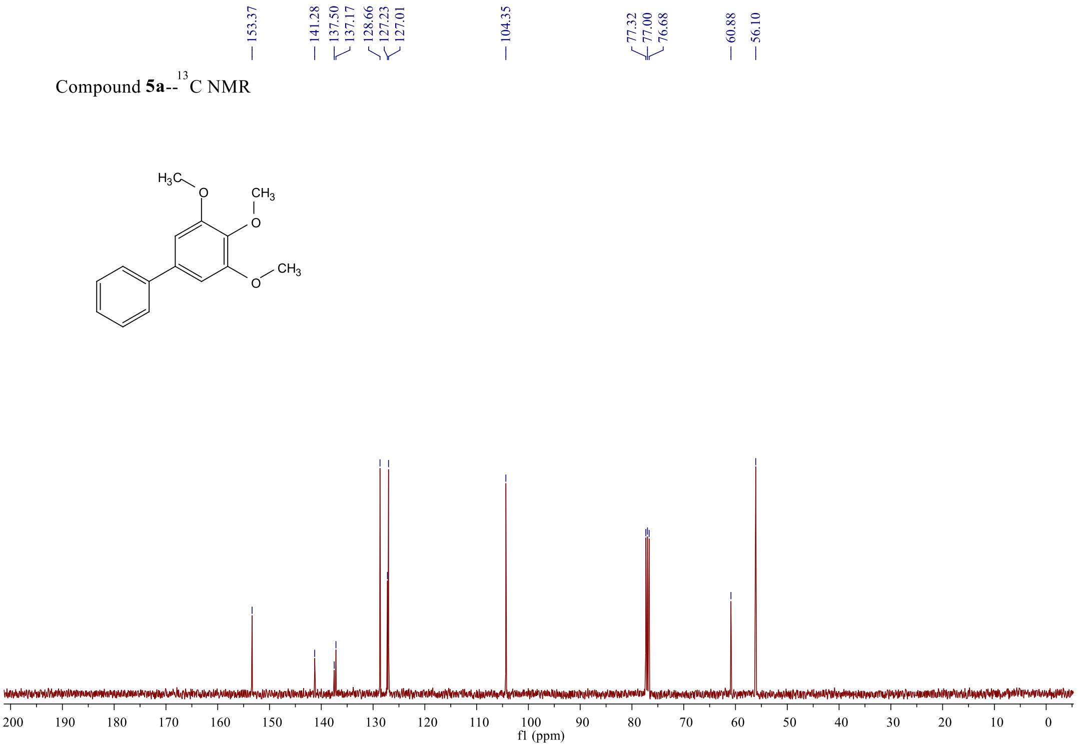

^{13}C NMR spectrum of compound **5a** (35 mg in 0.6 mL $CDCl_3$, 25 °C, Scan times ns = 512)

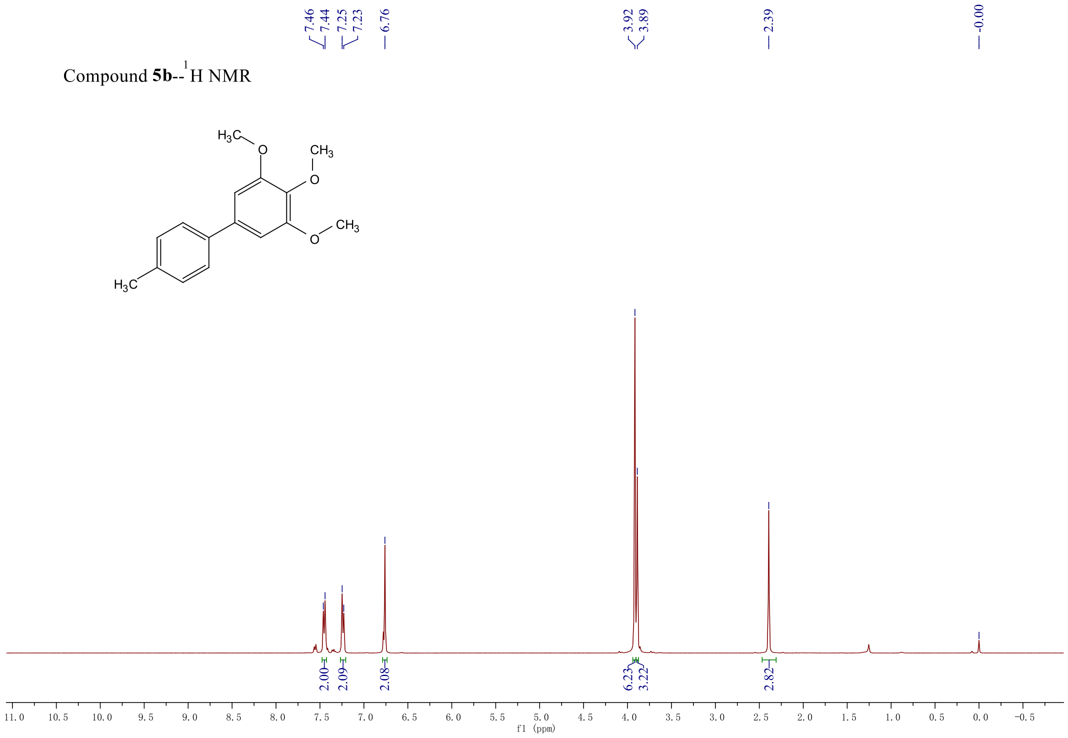

^{1}H NMR spectrum of compound **5b** (20 mg in 0.6 mL $CDCl_3$, 25 °C, Scan times ns = 32)

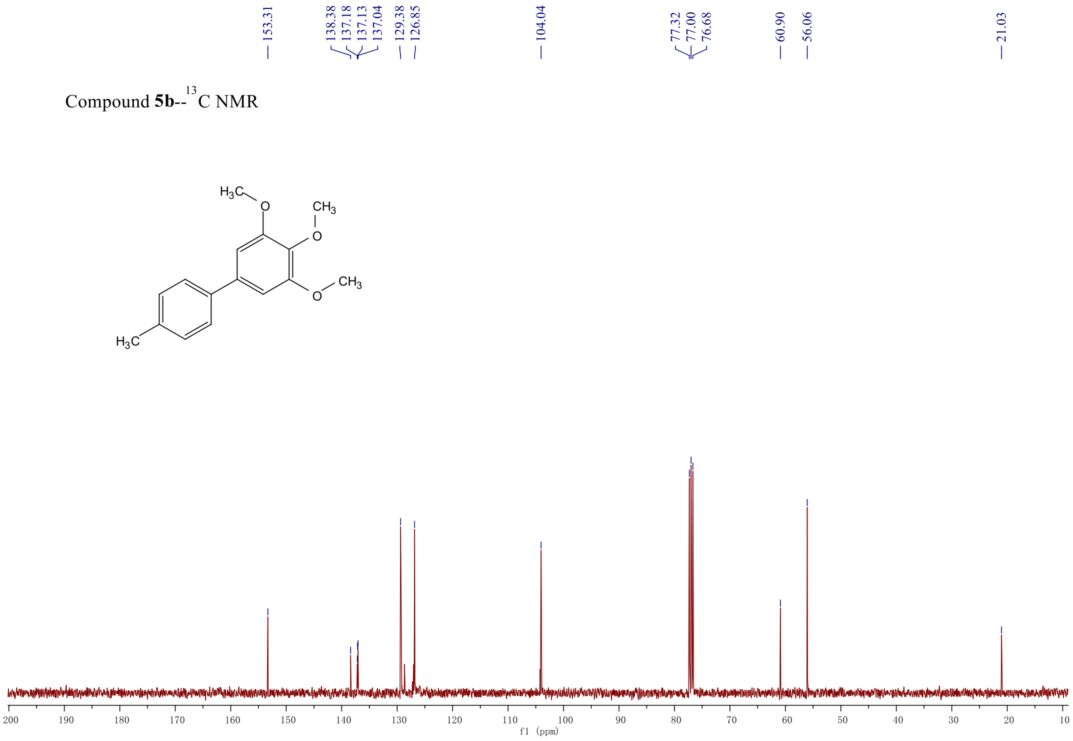

^{13}C NMR spectrum of compound **5b** (35 mg in 0.6 mL $CDCl_3$, 25 °C, Scan times ns = 512)

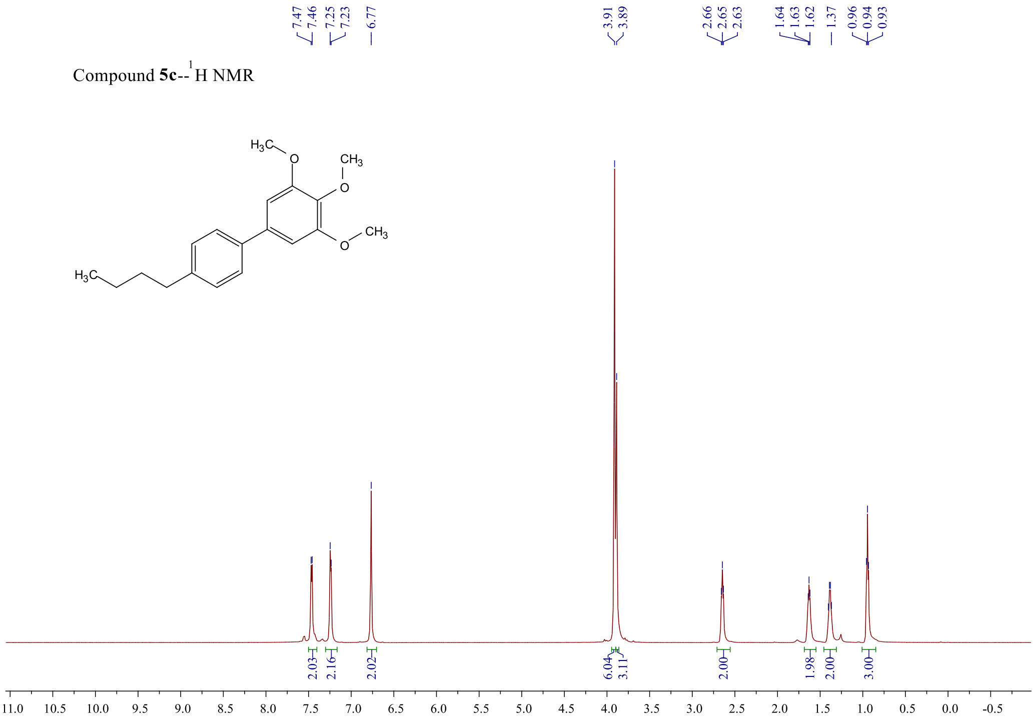

^{1}H NMR spectrum of compound **5c** (20 mg in 0.6 mL $CDCl_3$, 25 °C, Scan times ns = 32)

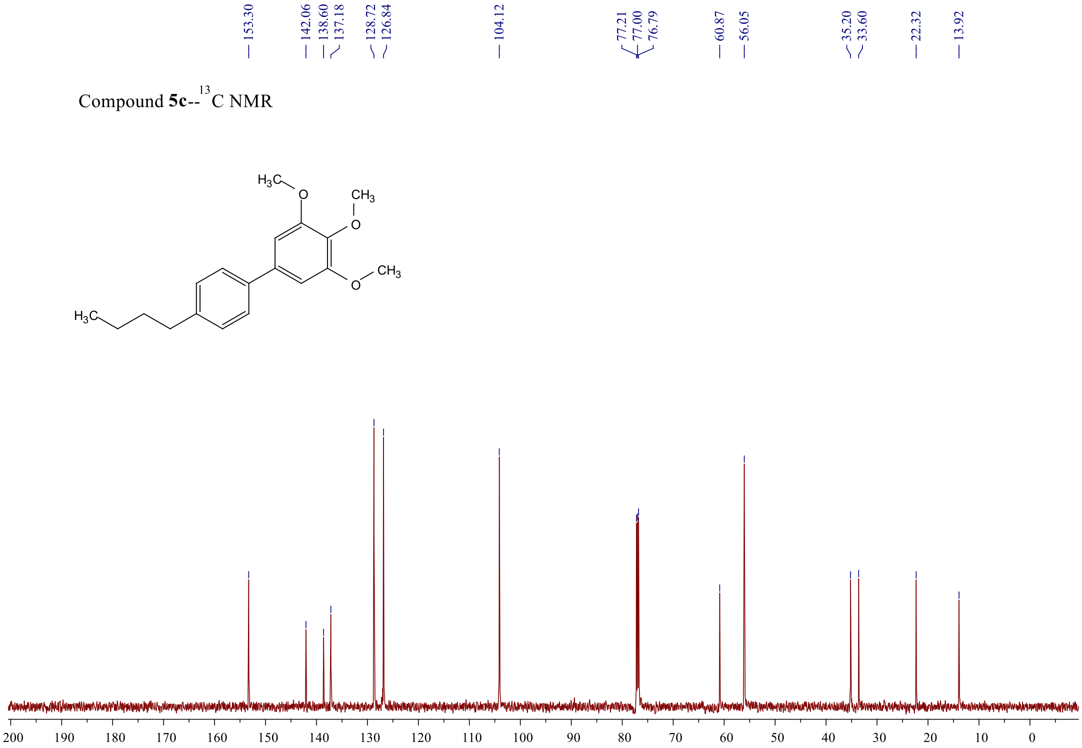

^{13}C NMR spectrum of compound **5c** (35 mg in 0.6 mL $CDCl_3$, 25 °C, Scan times ns = 512)

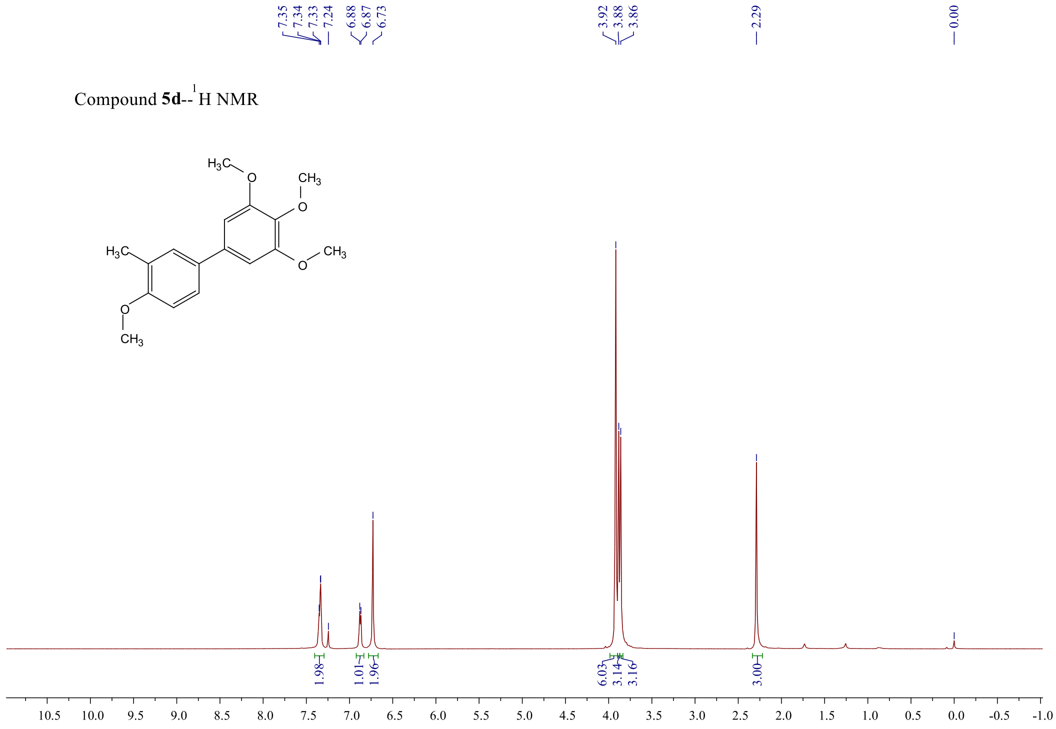

^{1}H NMR spectrum of compound **5d** (20 mg in 0.6 mL $CDCl_3$, 25 °C, Scan times ns = 32)

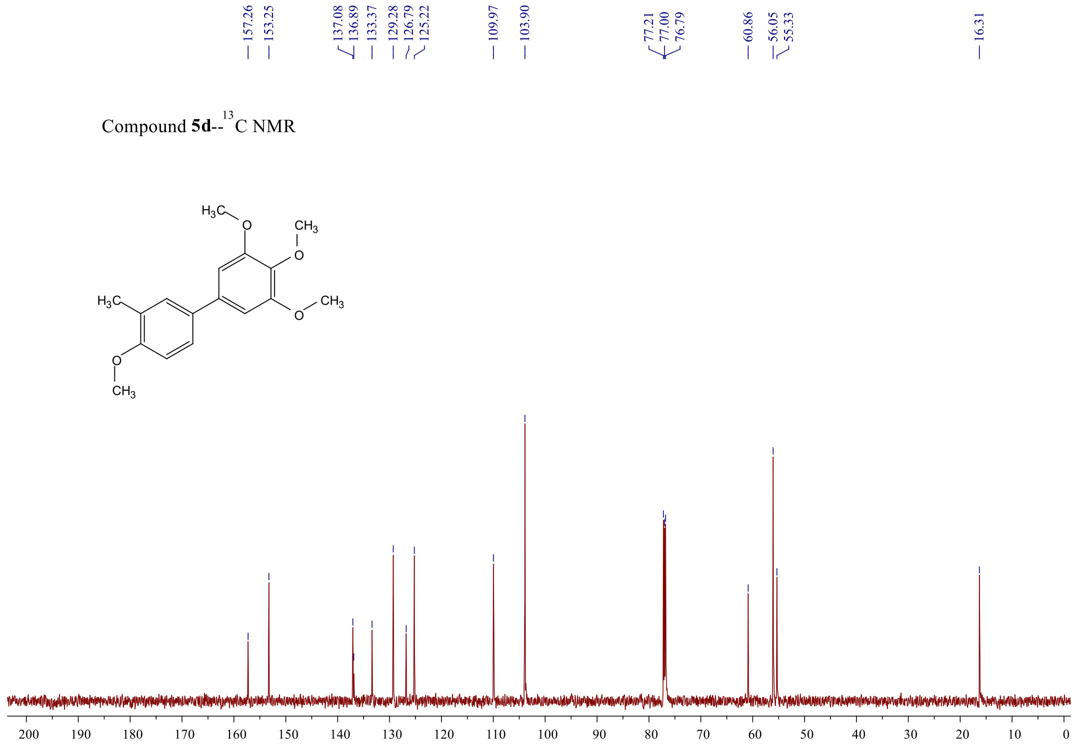

^{13}C NMR spectrum of compound **5d** (35 mg in 0.6 mL $CDCl_3$, 25 °C, Scan times ns = 512)

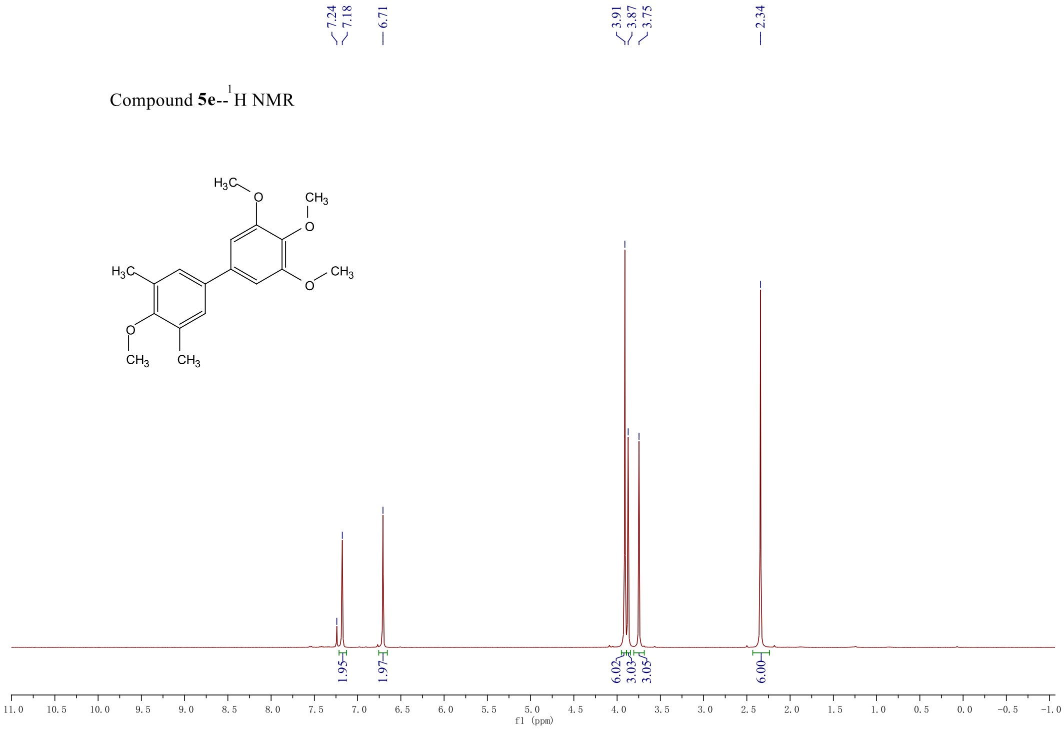

^{1}H NMR spectrum of compound **5e** (20 mg in 0.6 mL $CDCl_3$, 25 °C, Scan times ns = 32)

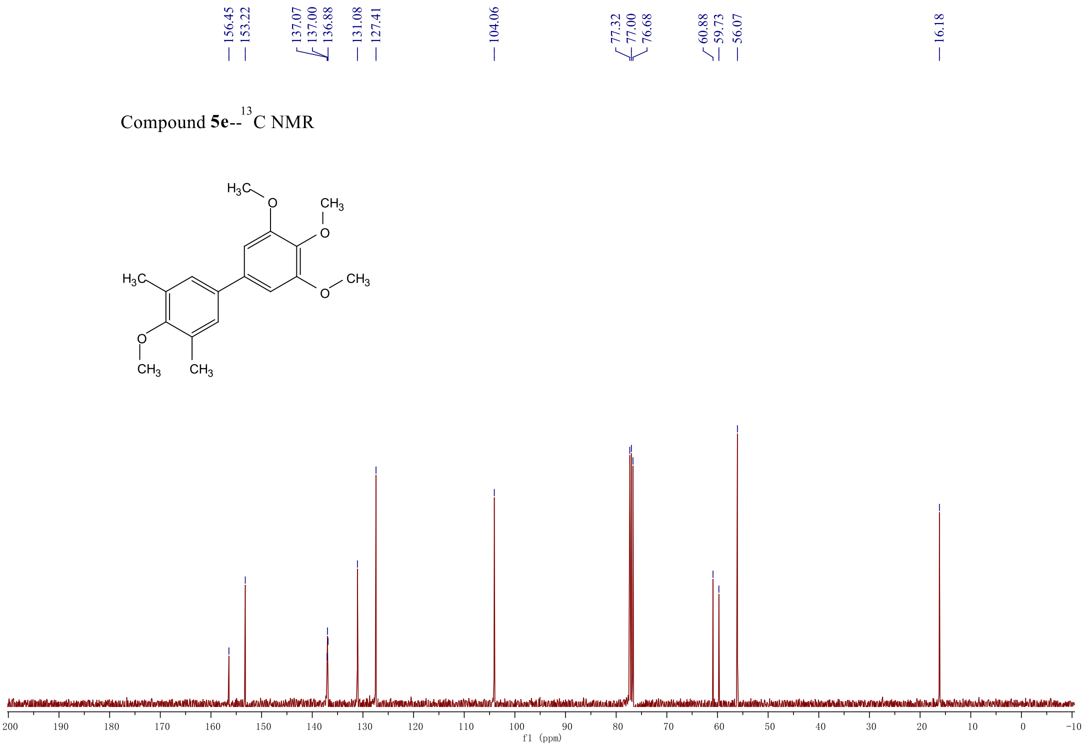

^{13}C NMR spectrum of compound **5e** (35 mg in 0.6 mL $CDCl_3$, 25 °C, Scan times ns = 512)

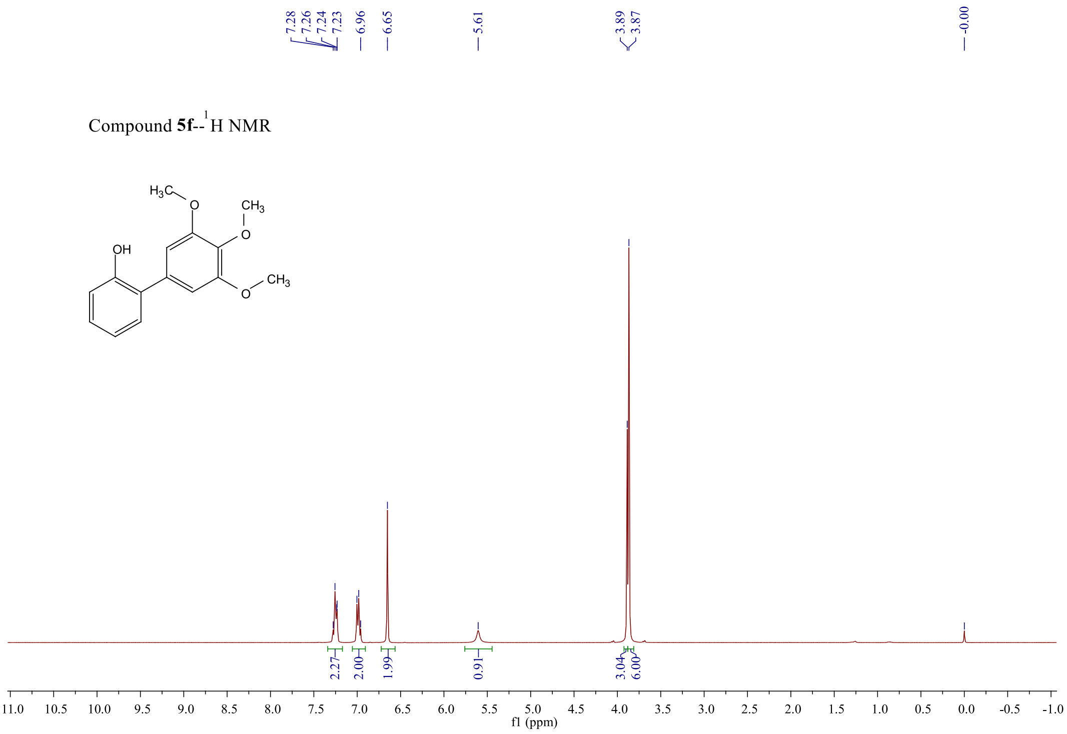

^{1}H NMR spectrum of compound **5f** (20 mg in 0.6 mL $CDCl_3$, 25 °C, Scan times ns = 32)

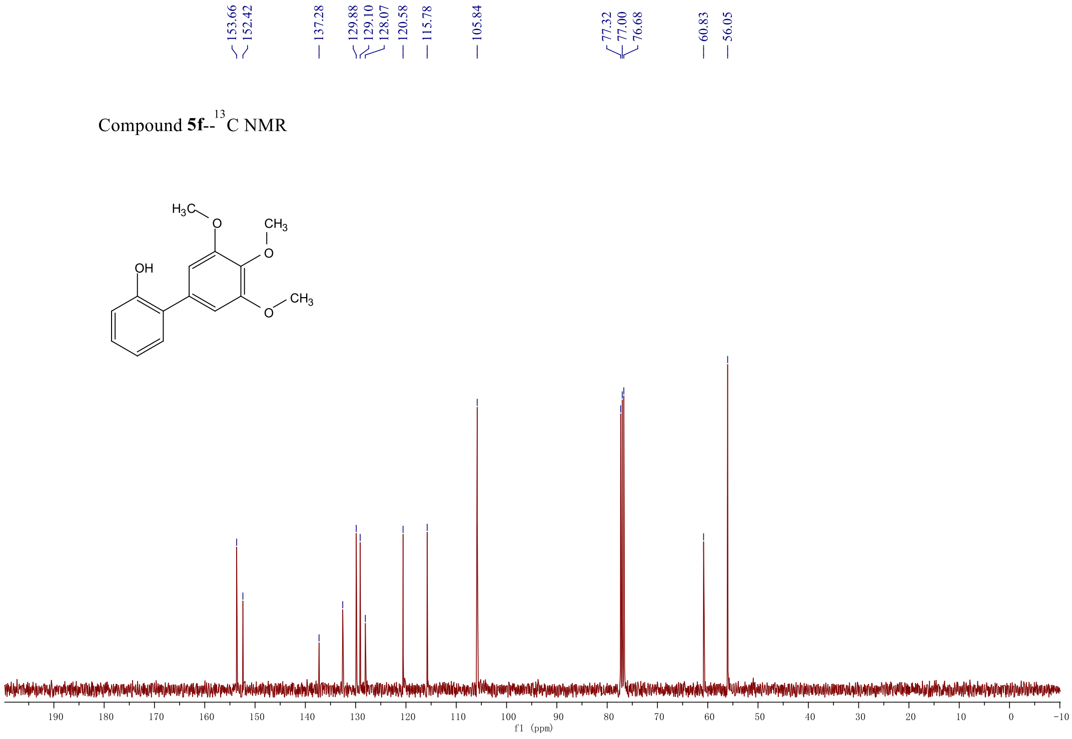

^{13}C NMR spectrum of compound **5f** (35 mg in 0.6 mL $CDCl_3$, 25 °C, Scan times ns = 512)

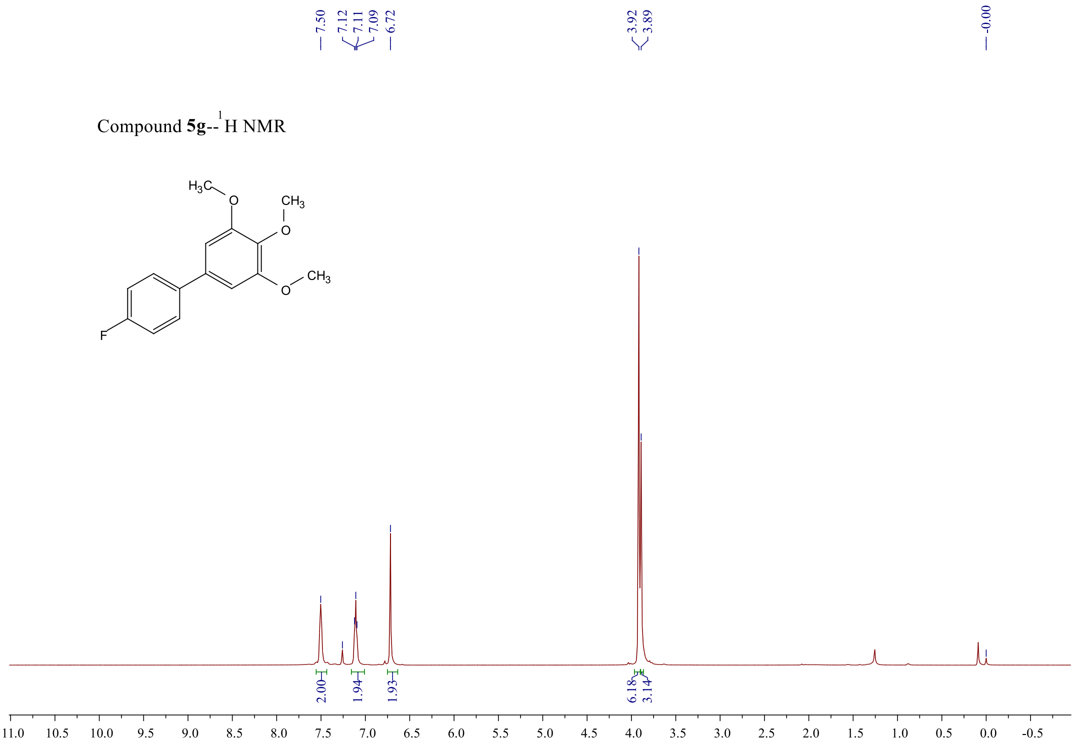

^{1}H NMR spectrum of compound **5g** (20 mg in 0.6 mL $CDCl_3$, 25 °C, Scan times ns = 32)

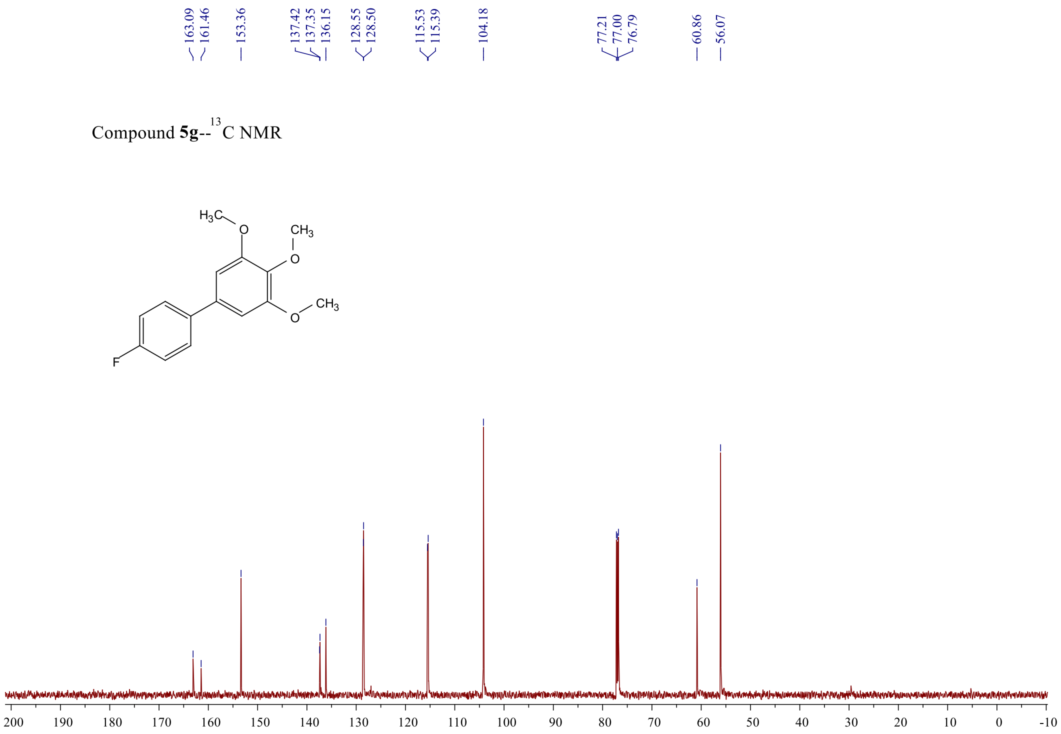

^{13}C NMR spectrum of compound **5g** (35 mg in 0.6 mL $CDCl_3$, 25 °C, Scan times ns = 512)

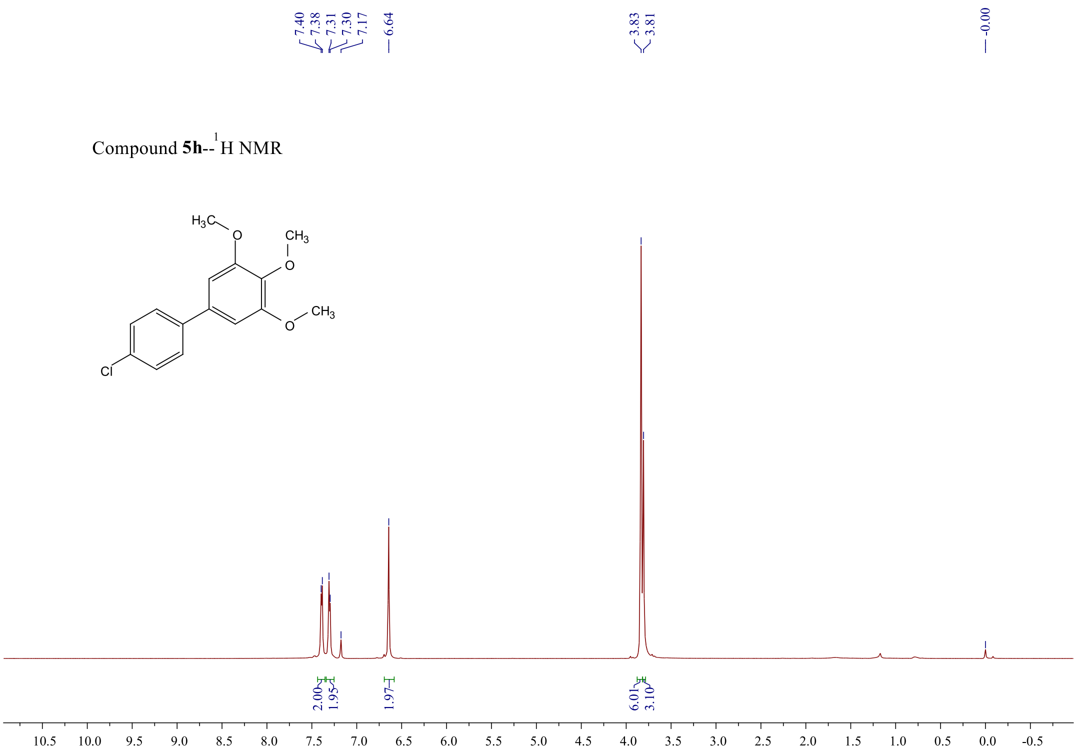

^{1}H NMR spectrum of compound **5h** (20 mg in 0.6 mL $CDCl_3$, 25 °C, Scan times ns = 32)

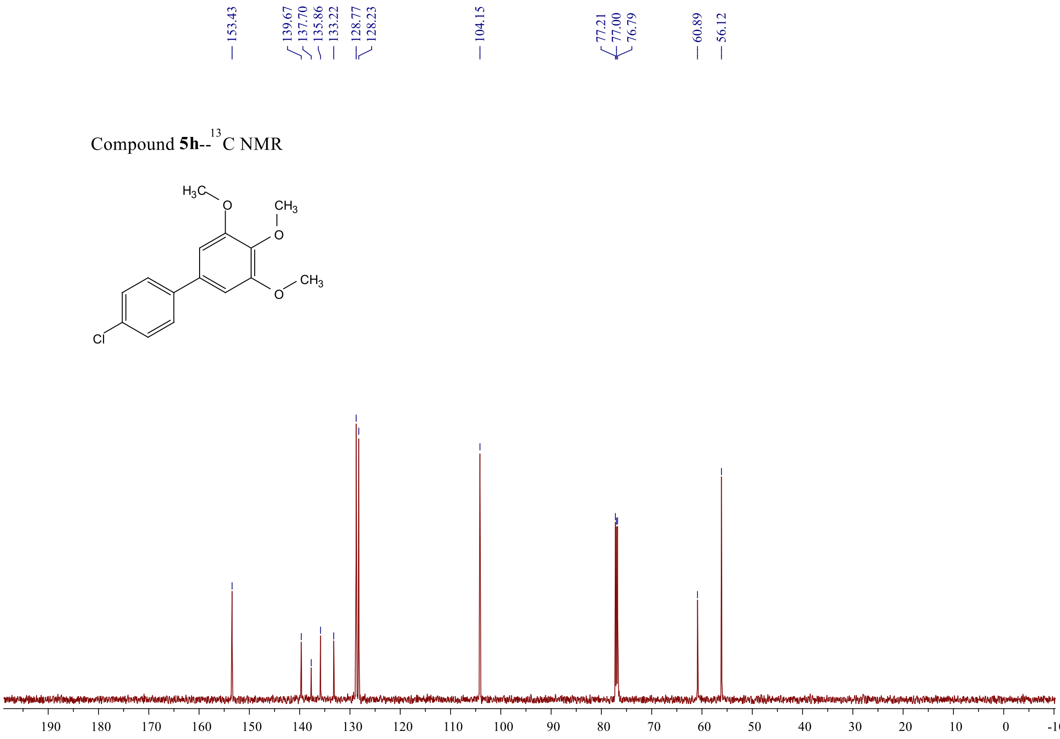

^{13}C NMR spectrum of compound **5h** (35 mg in 0.6 mL $CDCl_3$, 25 °C, Scan times ns = 512)

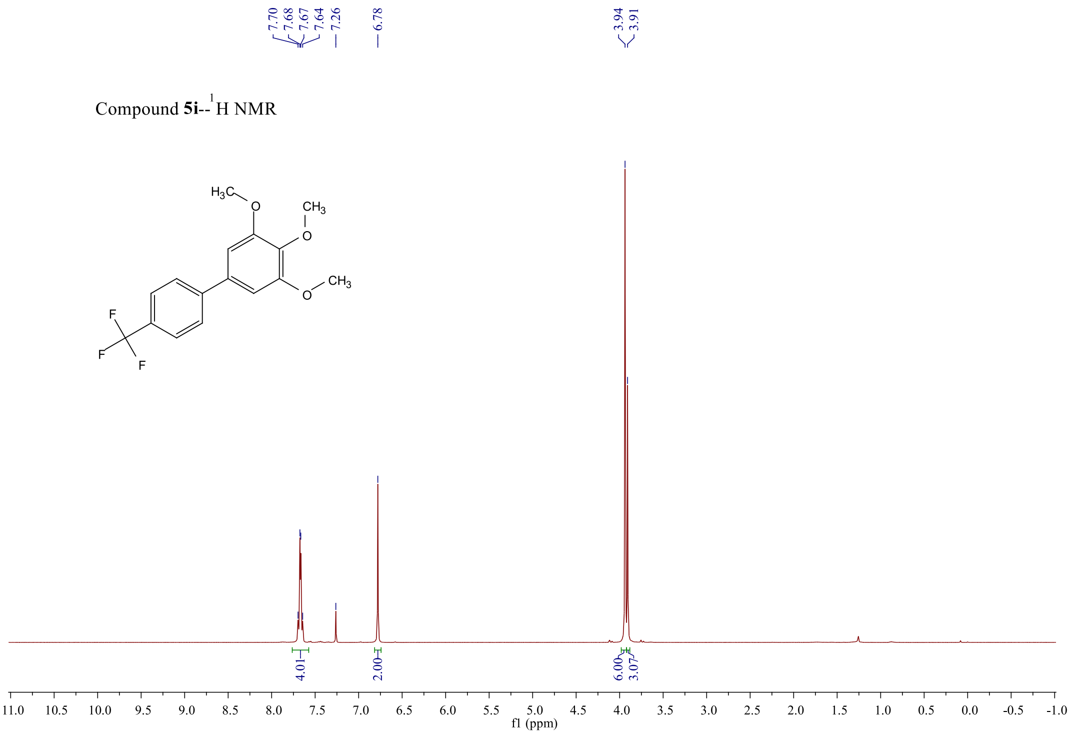

^{1}H NMR spectrum of compound **5i** (20 mg in 0.6 mL $CDCl_3$, 25 °C, Scan times ns = 32)

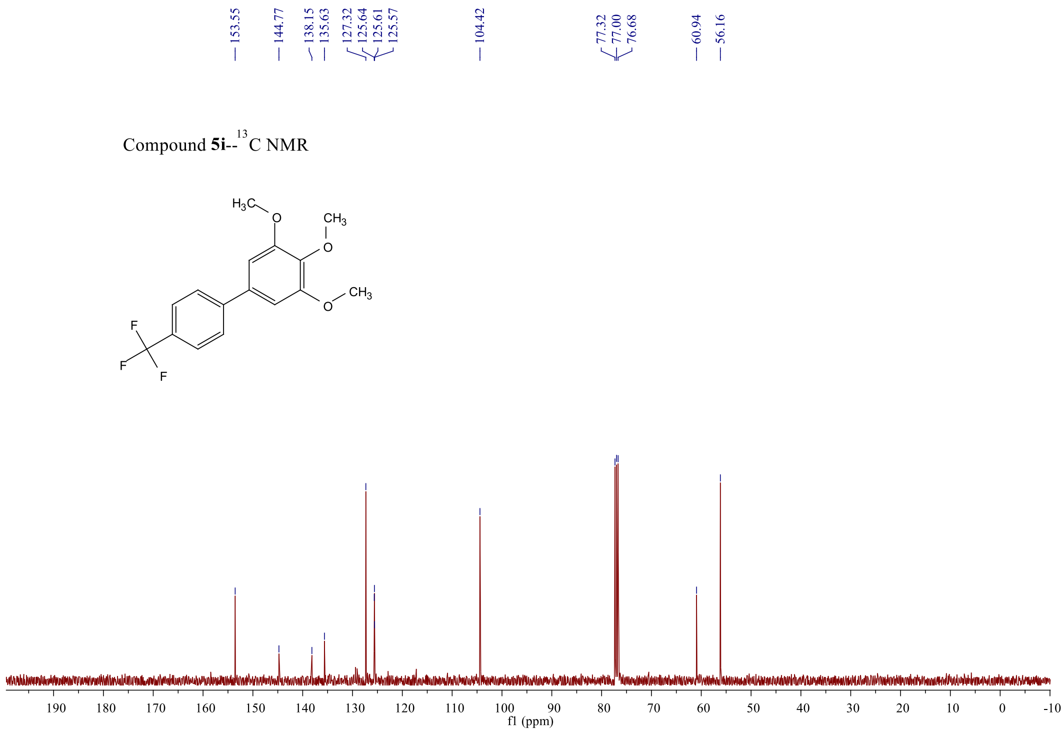

^{13}C NMR spectrum of compound **5i** (35 mg in 0.6 mL $CDCl_3$, 25 °C, Scan times ns = 512)

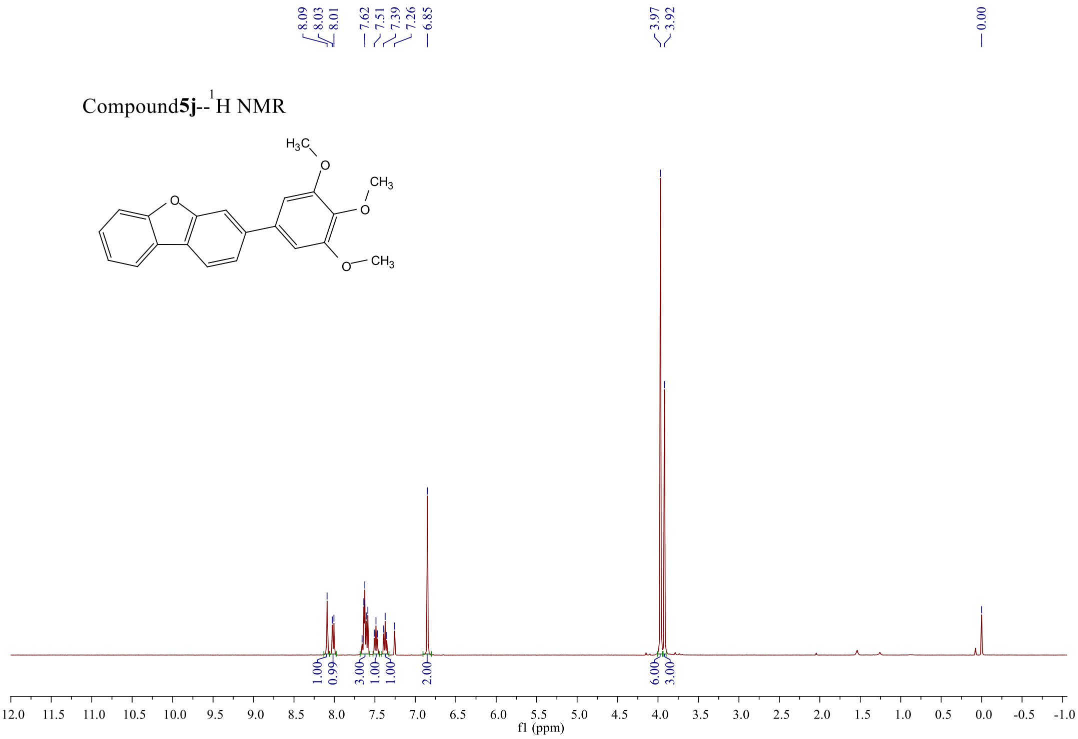

^{1}H NMR spectrum of compound **5j** (20 mg in 0.6 mL $CDCl_3$, 25 °C, Scan times ns = 32)

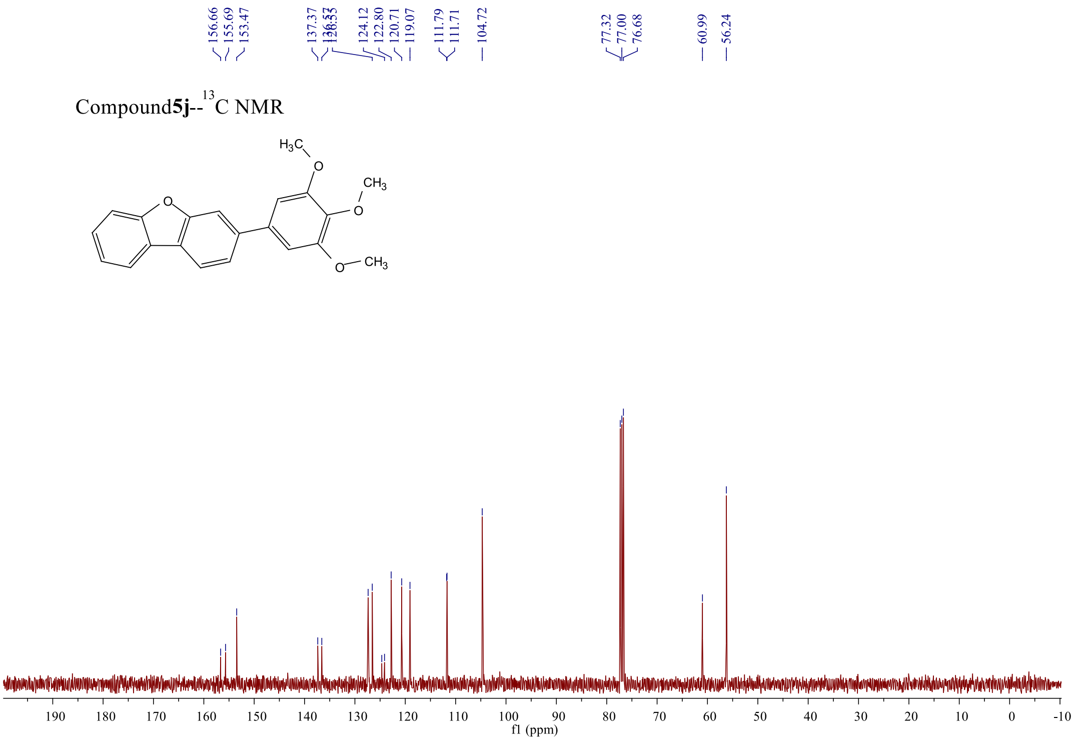

^{13}C NMR spectrum of compound **5j** (35 mg in 0.6 mL $CDCl_3$, 25 °C, Scan times ns = 512)

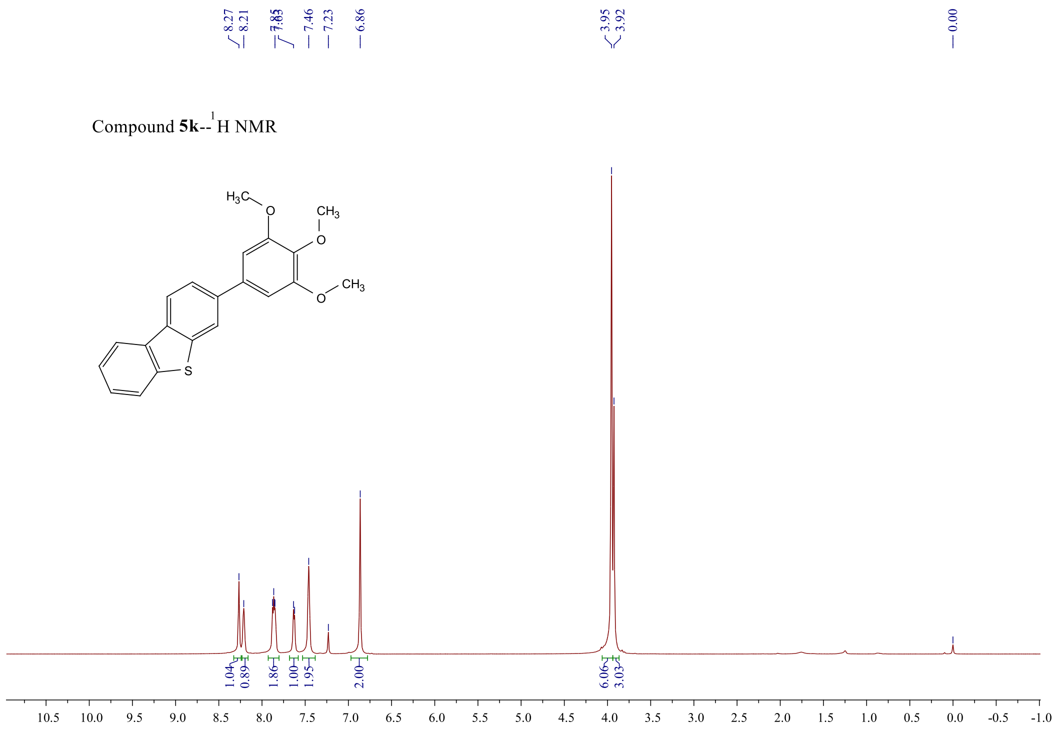

^{1}H NMR spectrum of compound **5k** (20 mg in 0.6 mL $CDCl_3$, 25 °C, Scan times ns = 32)

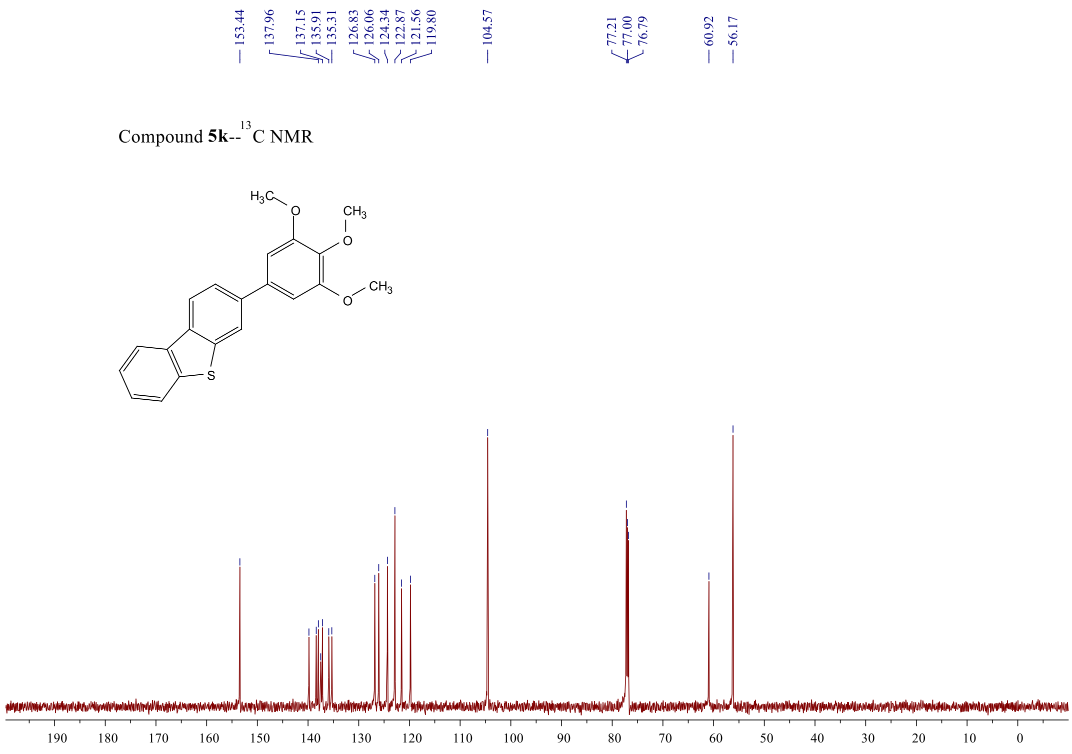

^{13}C NMR spectrum of compound **5k** (35 mg in 0.6 mL $CDCl_3$, 25 °C, Scan times ns = 512)

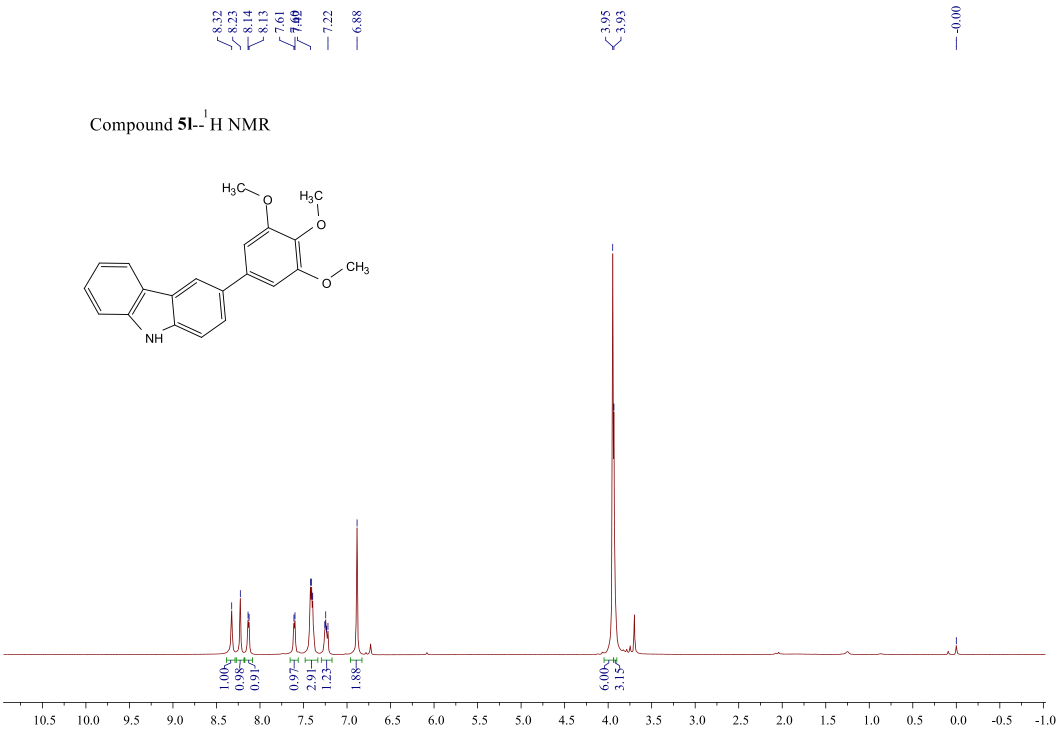

^{1}H NMR spectrum of compound **5l** (20 mg in 0.6 mL $CDCl_3$, 25 °C, Scan times ns = 32)

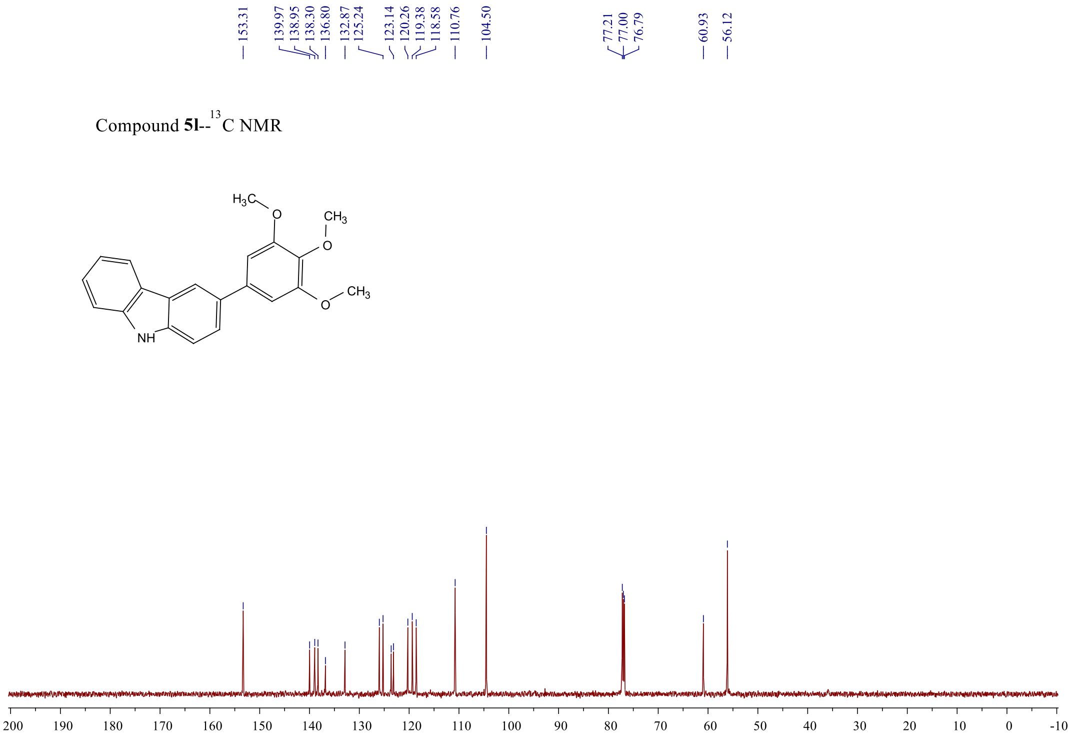

^{13}C NMR spectrum of compound **5l** (35 mg in 0.6 mL $CDCl_3$, 25 °C, Scan times ns = 512)

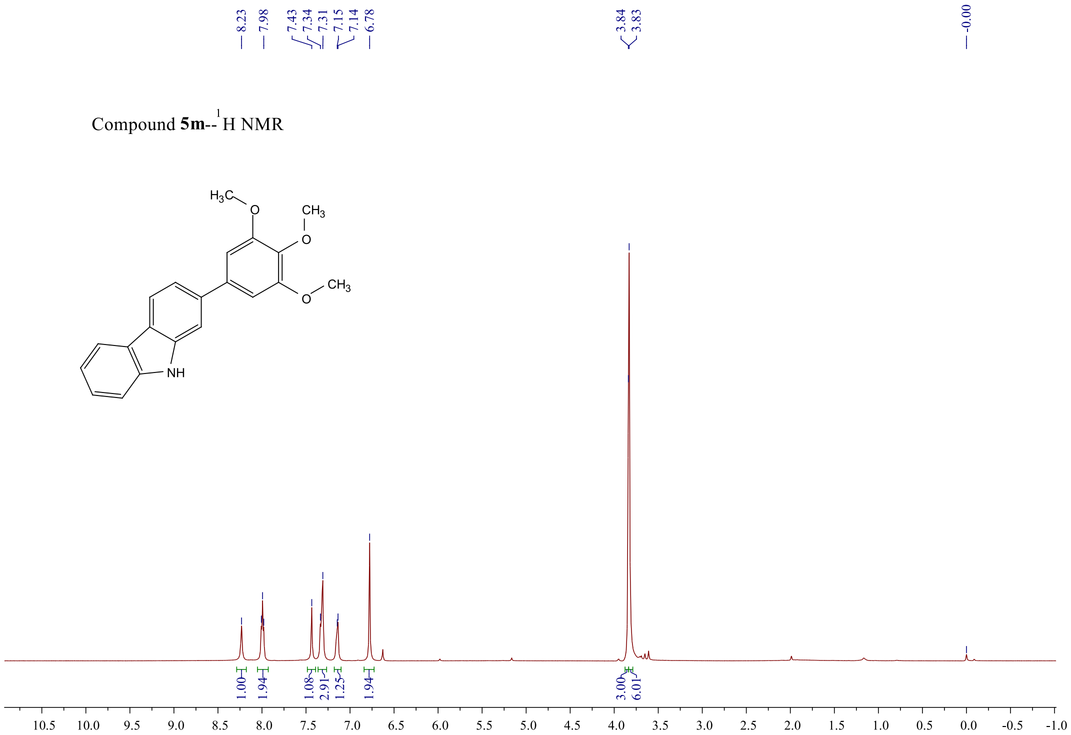

^{1}H NMR spectrum of compound **5m** (20 mg in 0.6 mL $CDCl_3$, 25 °C, Scan times ns = 32)

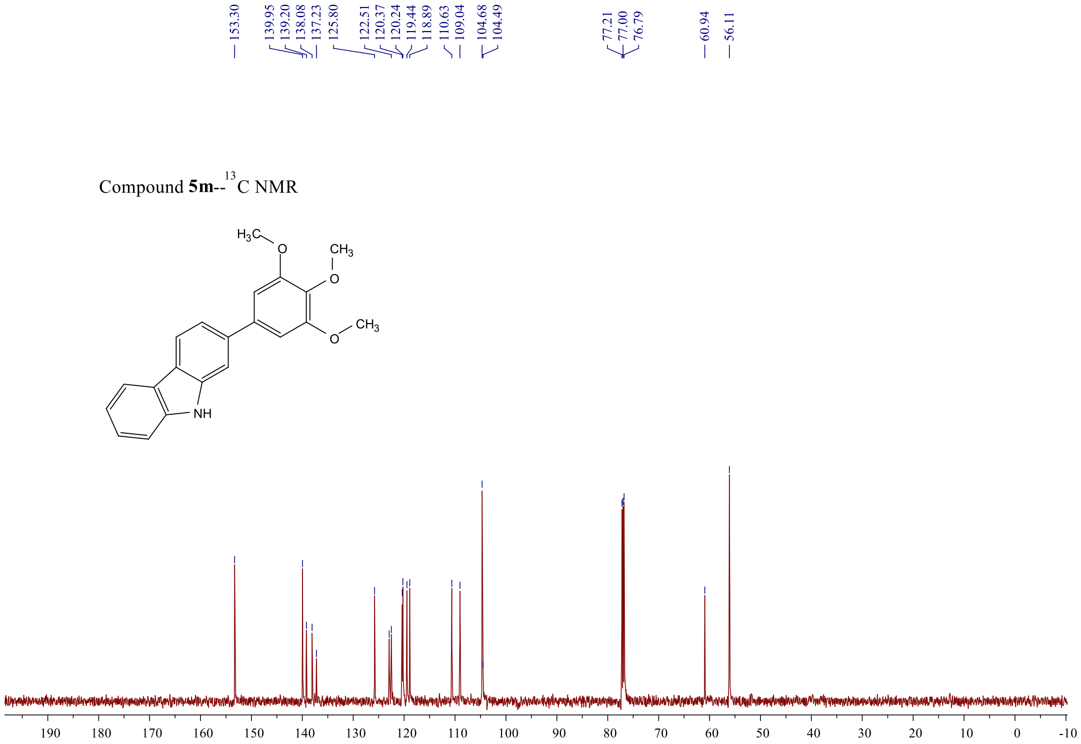

^{13}C NMR spectrum of compound **5m** (35 mg in 0.6 mL $CDCl_3$, 25 °C, Scan times ns = 512)

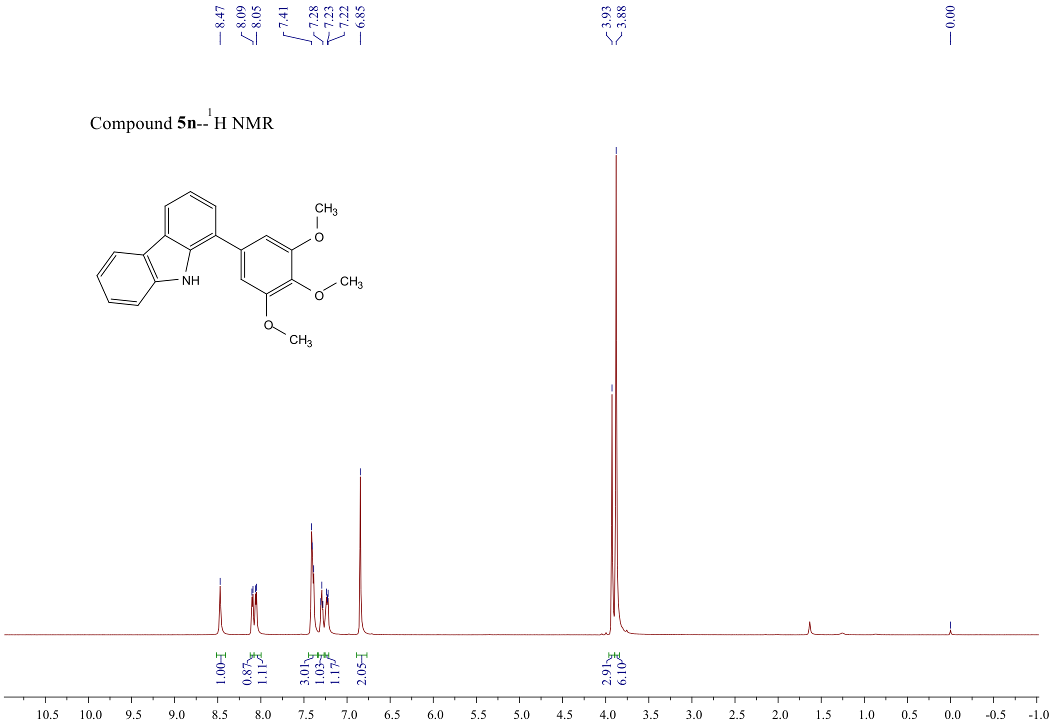

^{1}H NMR spectrum of compound **5n** (20 mg in 0.6 mL $CDCl_3$, 25 °C, Scan times ns = 32)

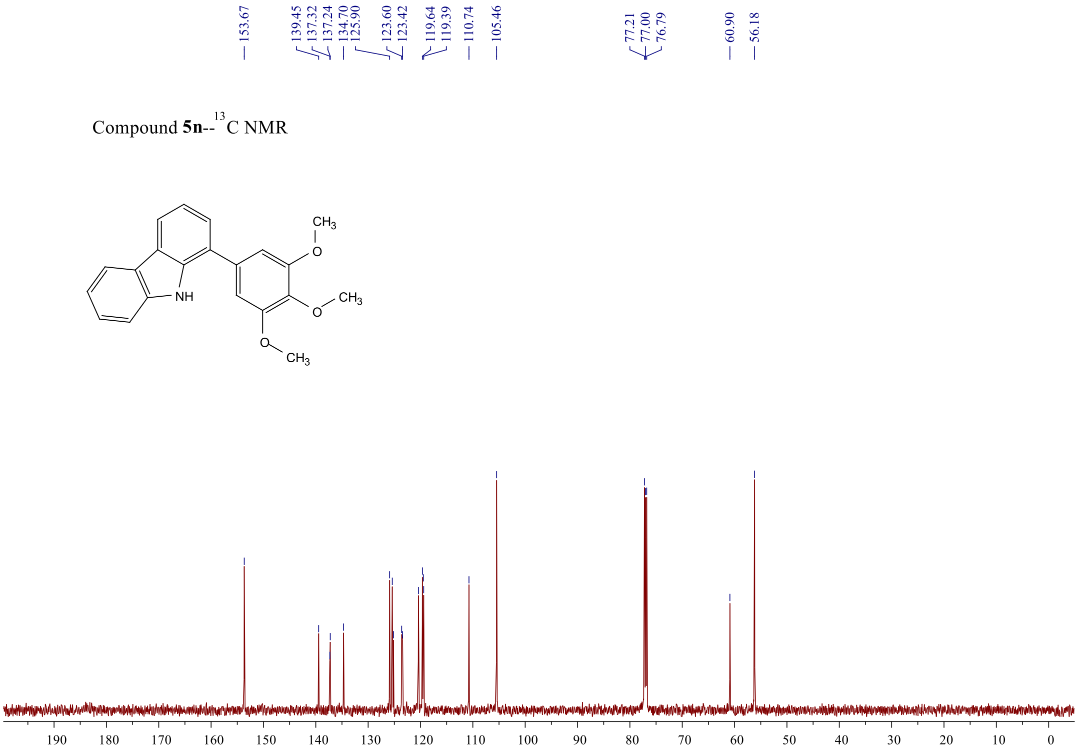

^{13}C NMR spectrum of compound **5n** (35 mg in 0.6 mL $CDCl_3$, 25 °C, Scan times ns = 512)

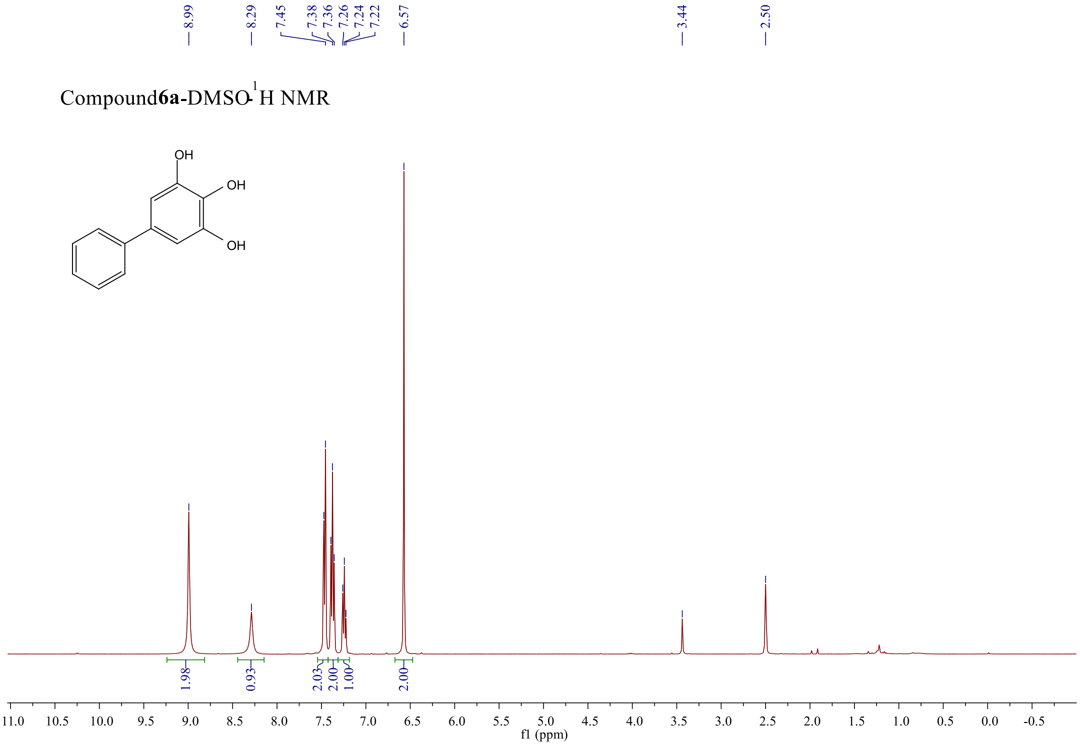

^{1}H NMR spectrum of compound **6a** (20 mg in 0.6 mL DMSO-d_6, 25 °C, Scan times ns = 32)

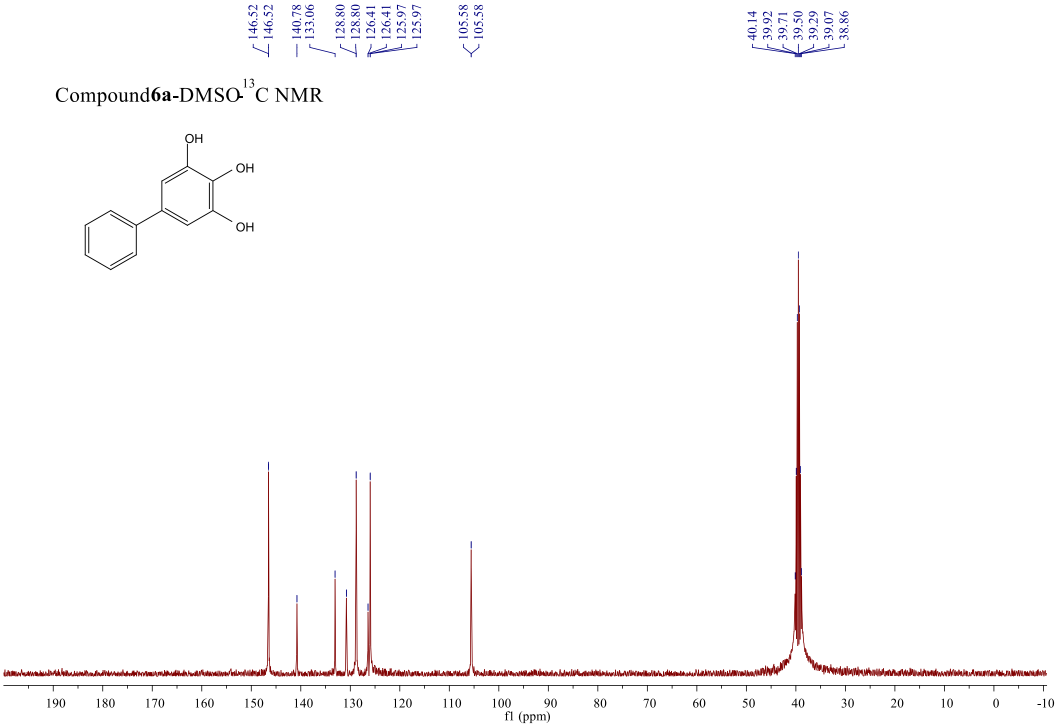

^{13}C NMR spectrum of compound **6a** (35 mg in 0.6 mL DMSO-d_6, 25 °C, Scan times ns = 1024)

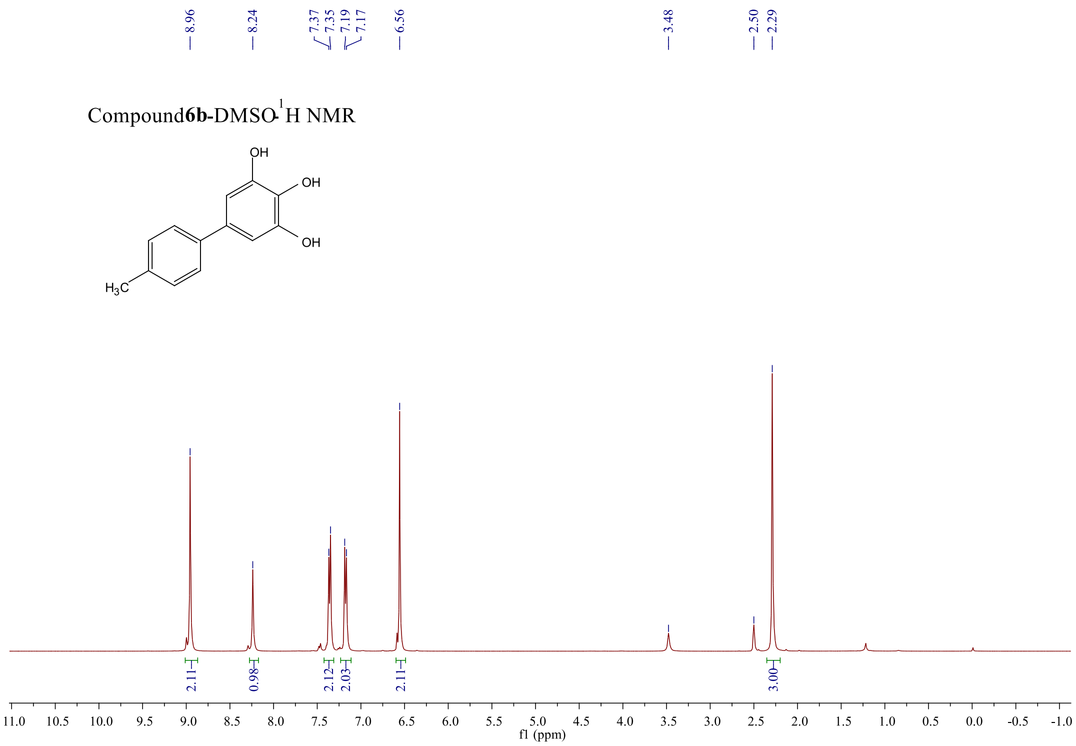

^{1}H NMR spectrum of compound **6b** (20 mg in 0.6 mL DMSO-d_6, 25 °C, Scan times ns = 32)

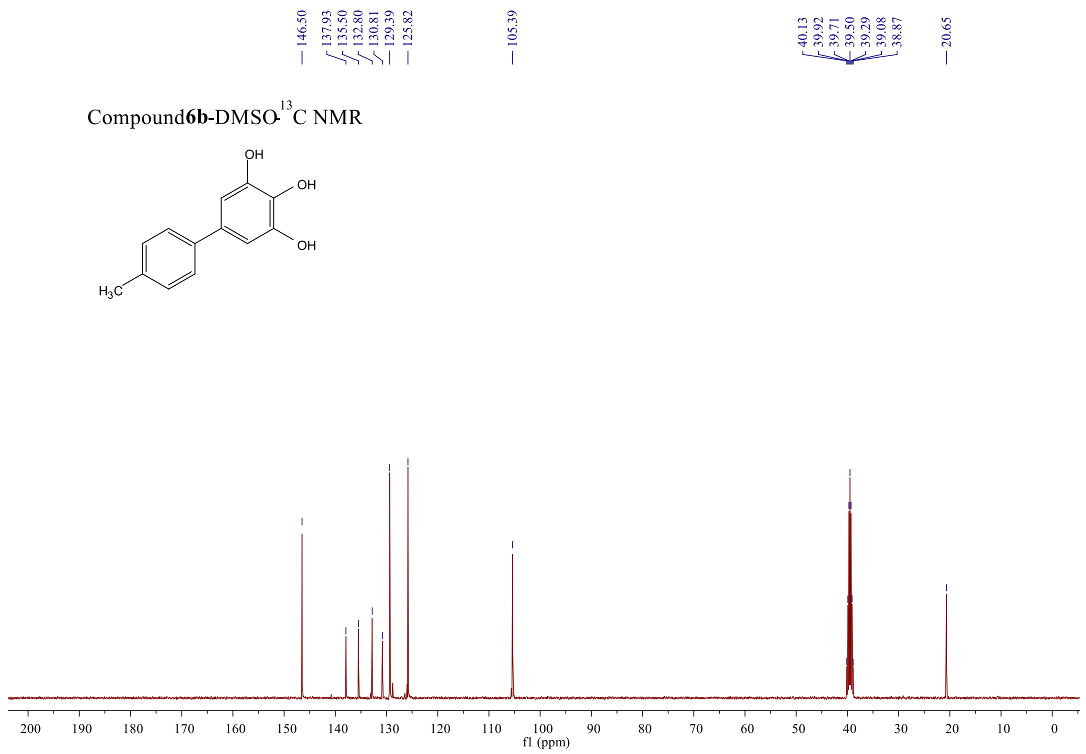

^{13}C NMR spectrum of compound **6b** (35 mg in 0.6 mL DMSO-d_6, 25 °C, Scan times ns = 1024)

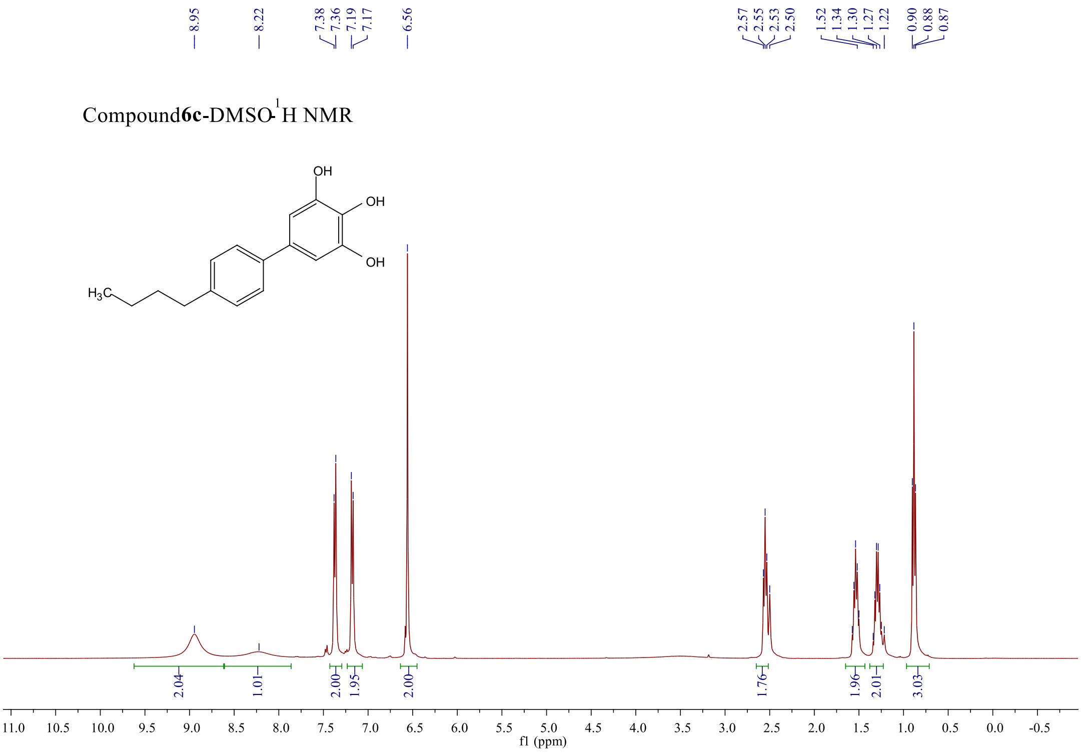

^{1}H NMR spectrum of compound **6c** (20 mg in 0.6 mL DMSO-d_6, 25 °C, Scan times ns = 32)

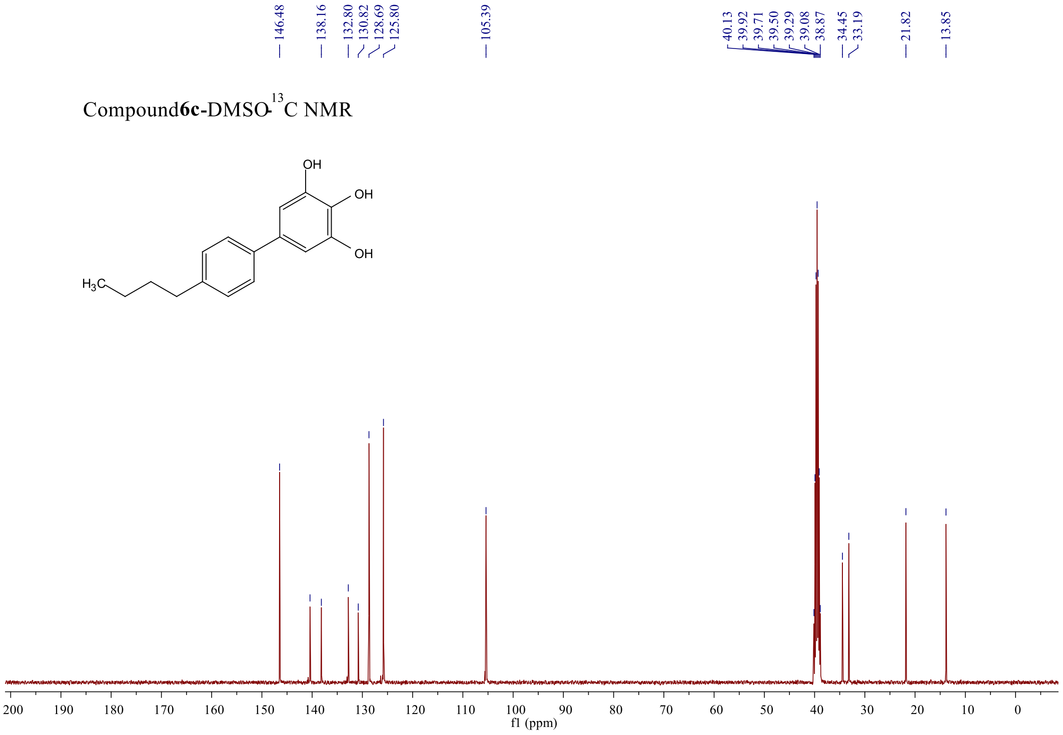

^{13}C NMR spectrum of compound **6c** (35 mg in 0.6 mL DMSO-d_6, 25 °C, Scan times ns = 1024)

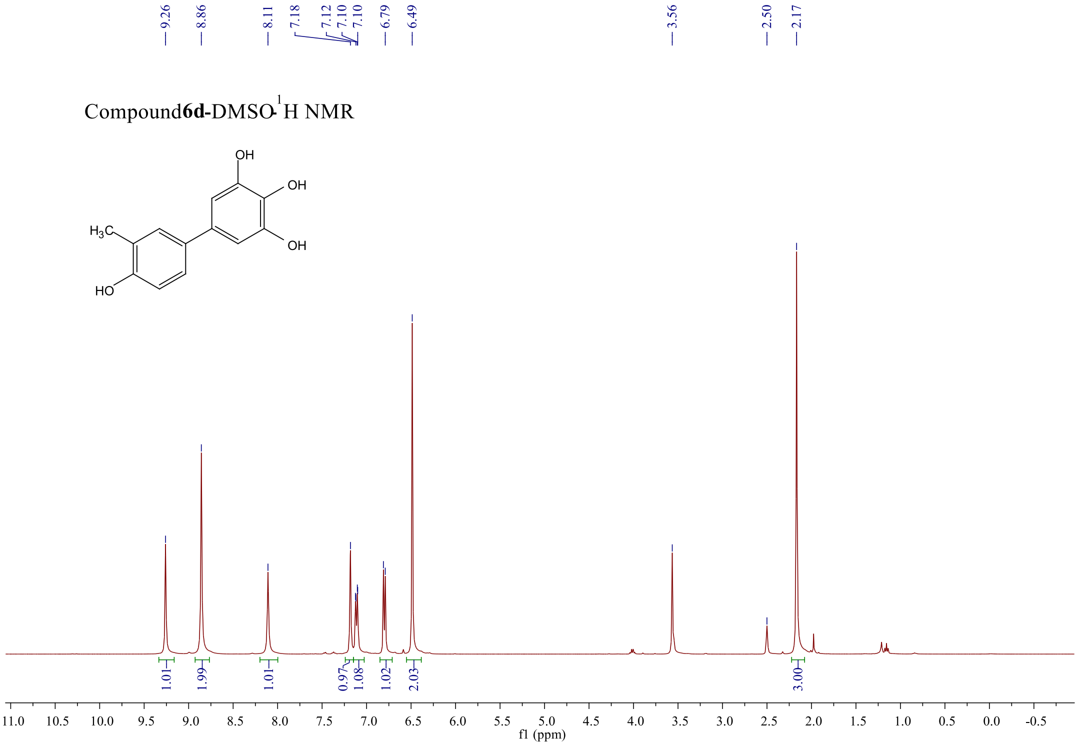

^{1}H NMR spectrum of compound **6d** (20 mg in 0.6 mL DMSO-d_6, 25 °C, Scan times ns = 32)

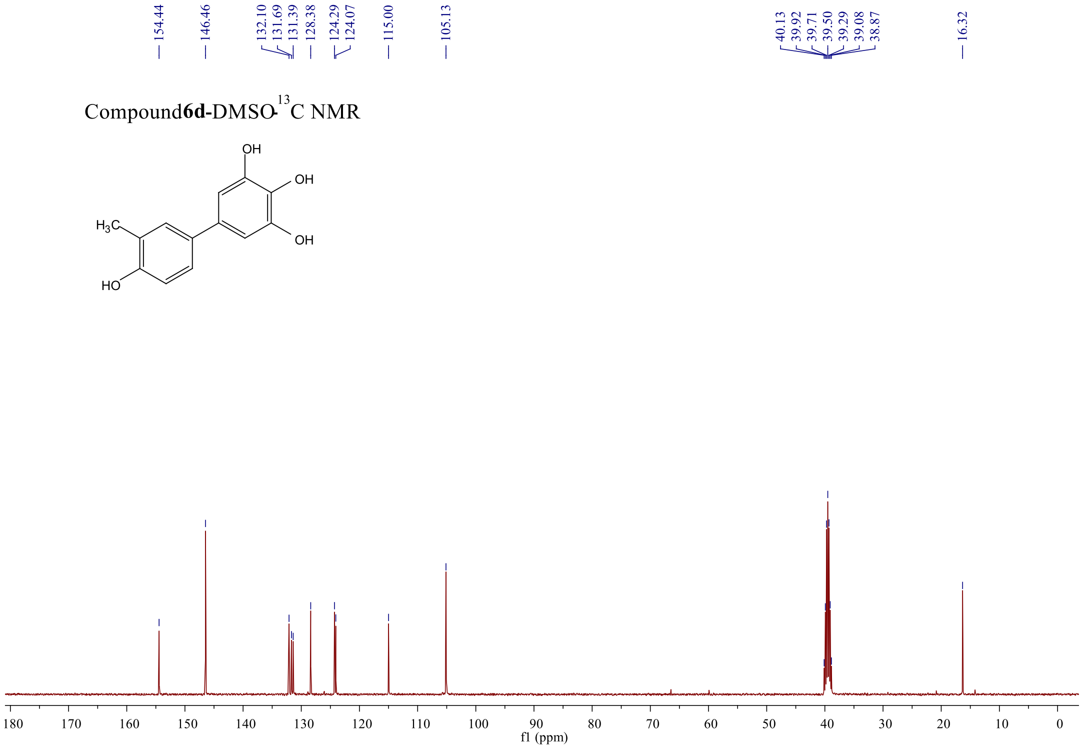

^{13}C NMR spectrum of compound **6d** (35 mg in 0.6 mL DMSO-d_6, 25 °C, Scan times ns = 1024)

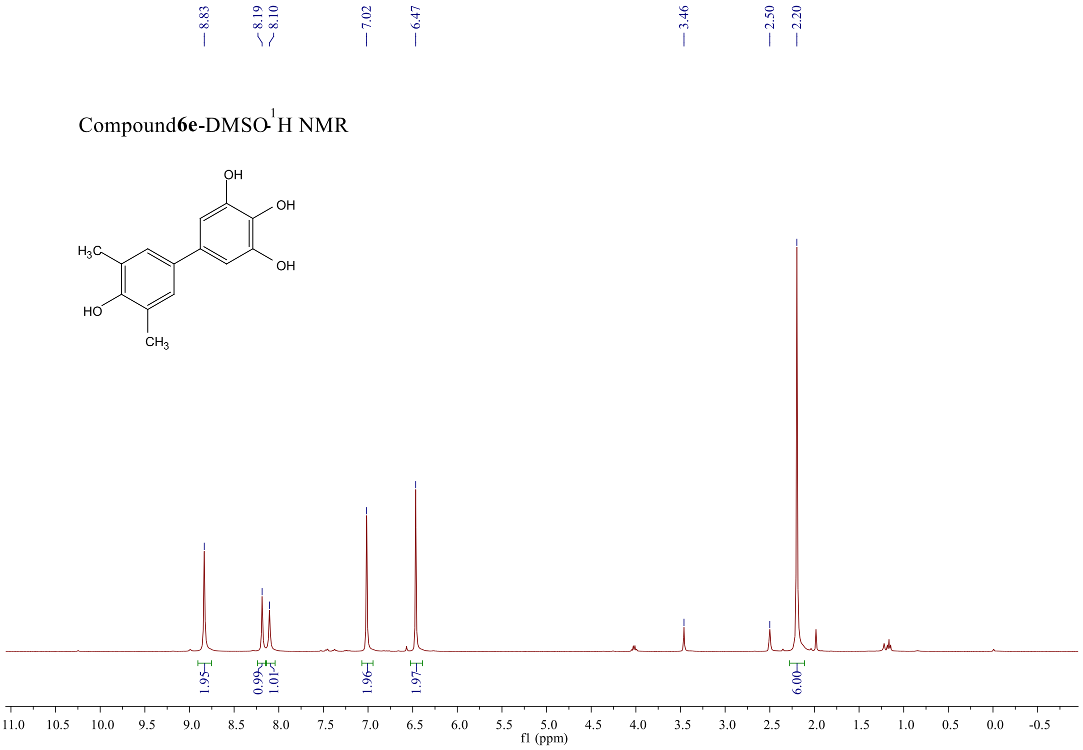

^{1}H NMR spectrum of compound **6e** (20 mg in 0.6 mL DMSO-d_6, 25 °C, Scan times ns = 32)

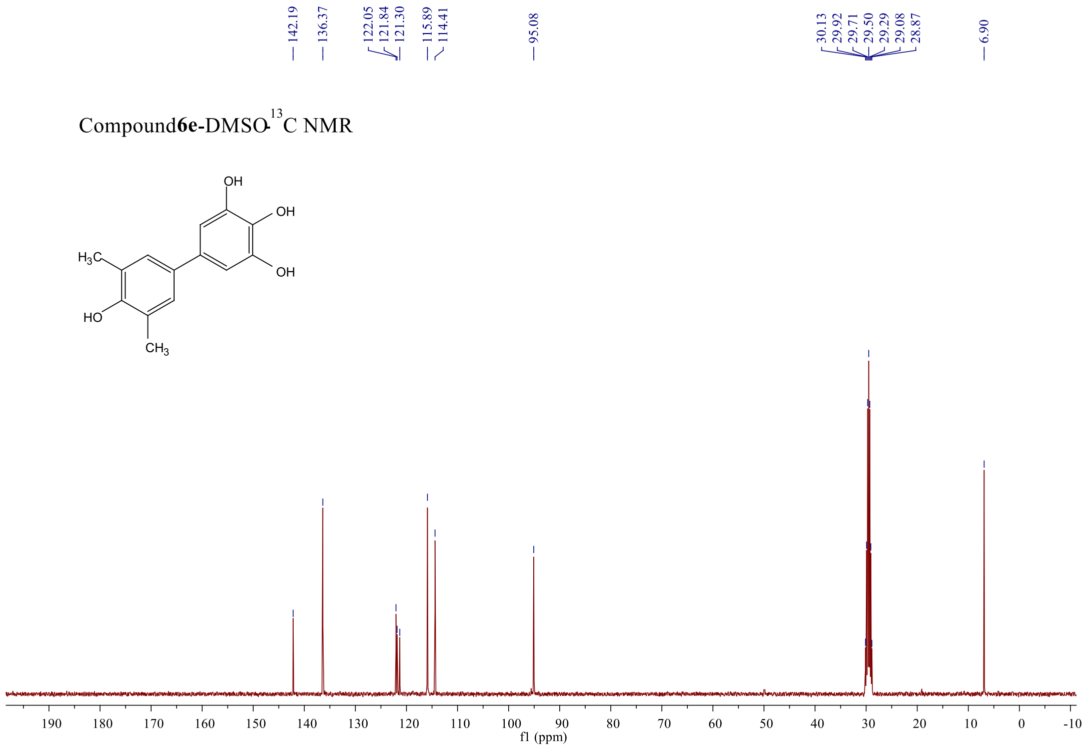

^{13}C NMR spectrum of compound **6e** (35 mg in 0.6 mL DMSO-d_6, 25 °C, Scan times ns = 1024)

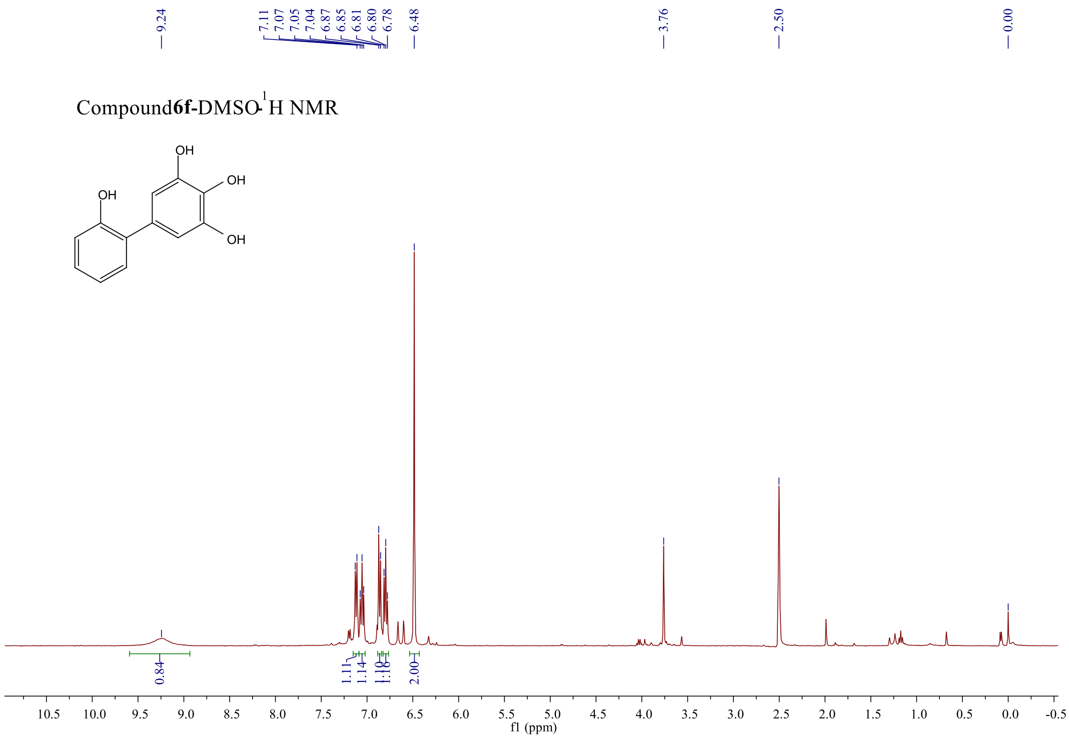

^{1}H NMR spectrum of compound **6f** (20 mg in 0.6 mL DMSO-d_6, 25 °C, Scan times ns = 32)

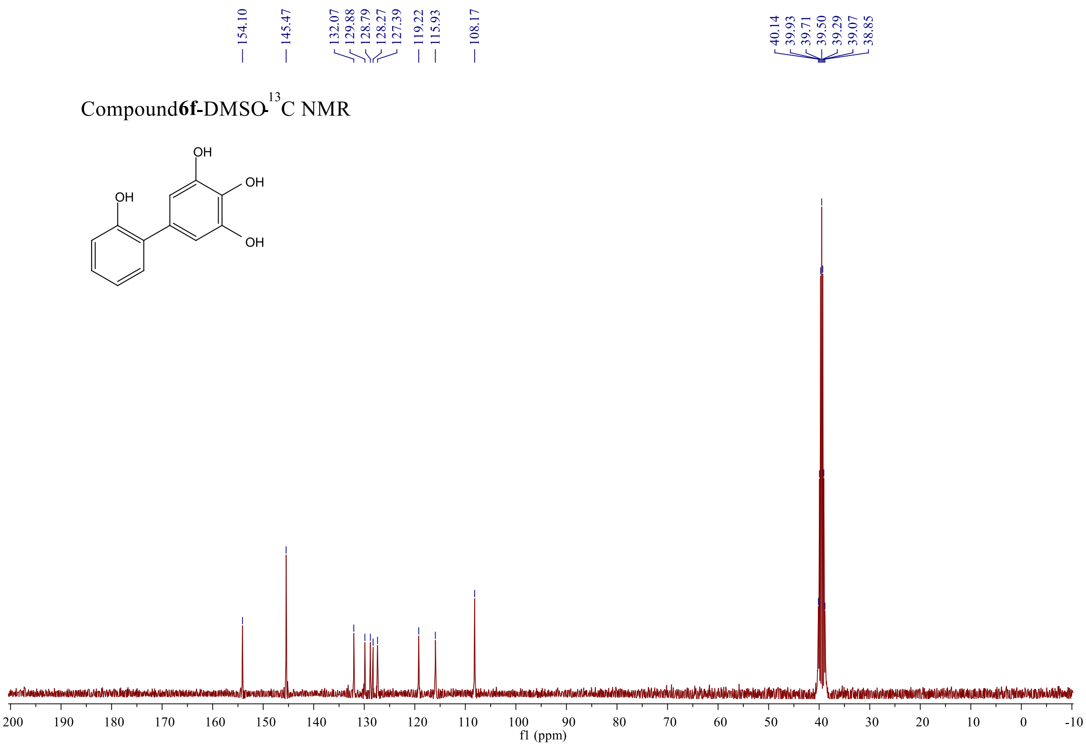

^{13}C NMR spectrum of compound **6f** (35 mg in 0.6 mL DMSO-d_6, 25 °C, Scan times ns = 1024)

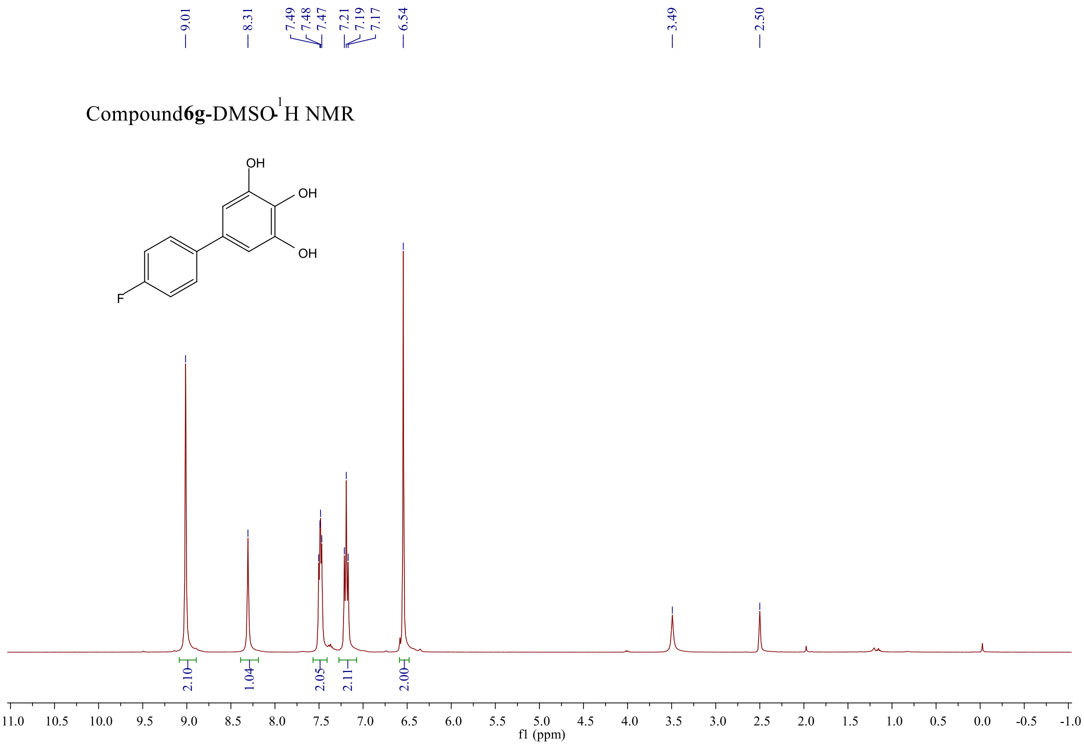

^{1}H NMR spectrum of compound **6g** (20 mg in 0.6 mL DMSO-d_6, 25 °C, Scan times ns = 32)

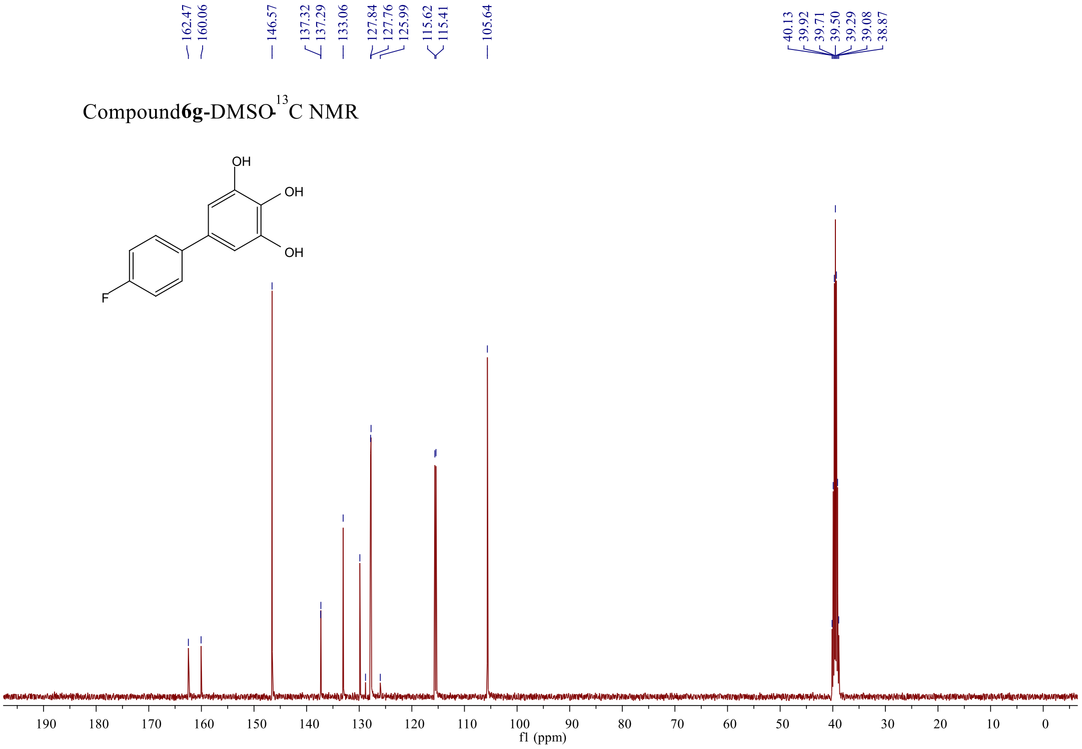

^{13}C NMR spectrum of compound **6g** (35 mg in 0.6 mL DMSO-d_6, 25 °C, Scan times ns = 1024)

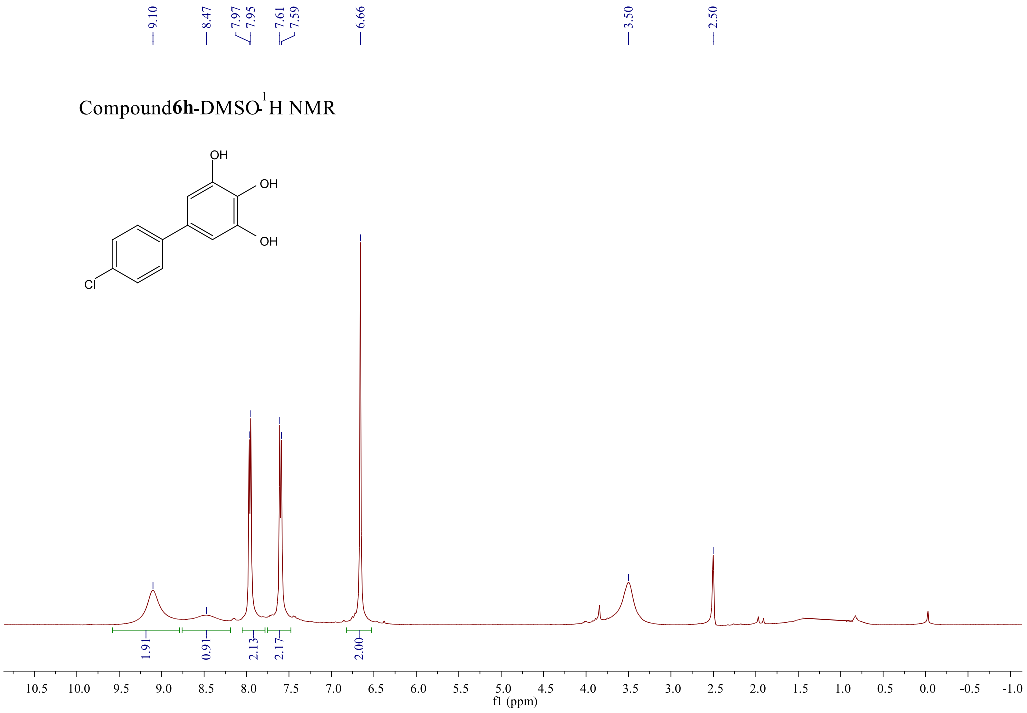

^{1}H NMR spectrum of compound **6h** (20 mg in 0.6 mL DMSO-d_6, 25 °C, Scan times ns = 32)

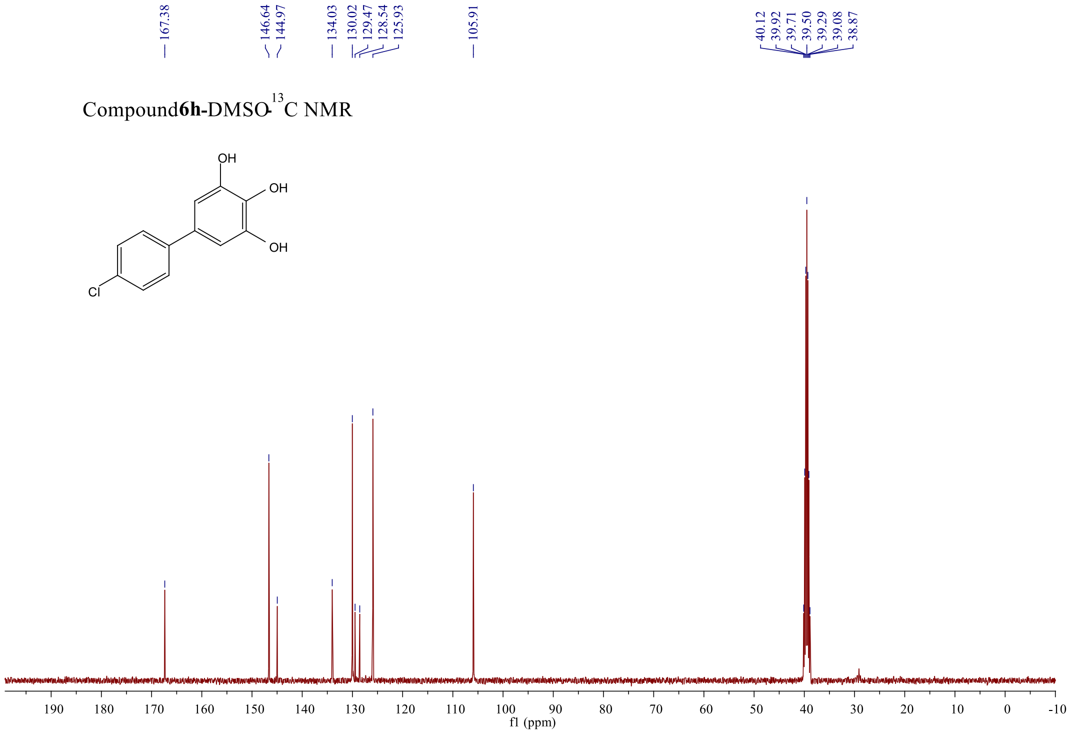

^{13}C NMR spectrum of compound **6h** (35 mg in 0.6 mL DMSO-d_6, 25 °C, Scan times ns = 1024)

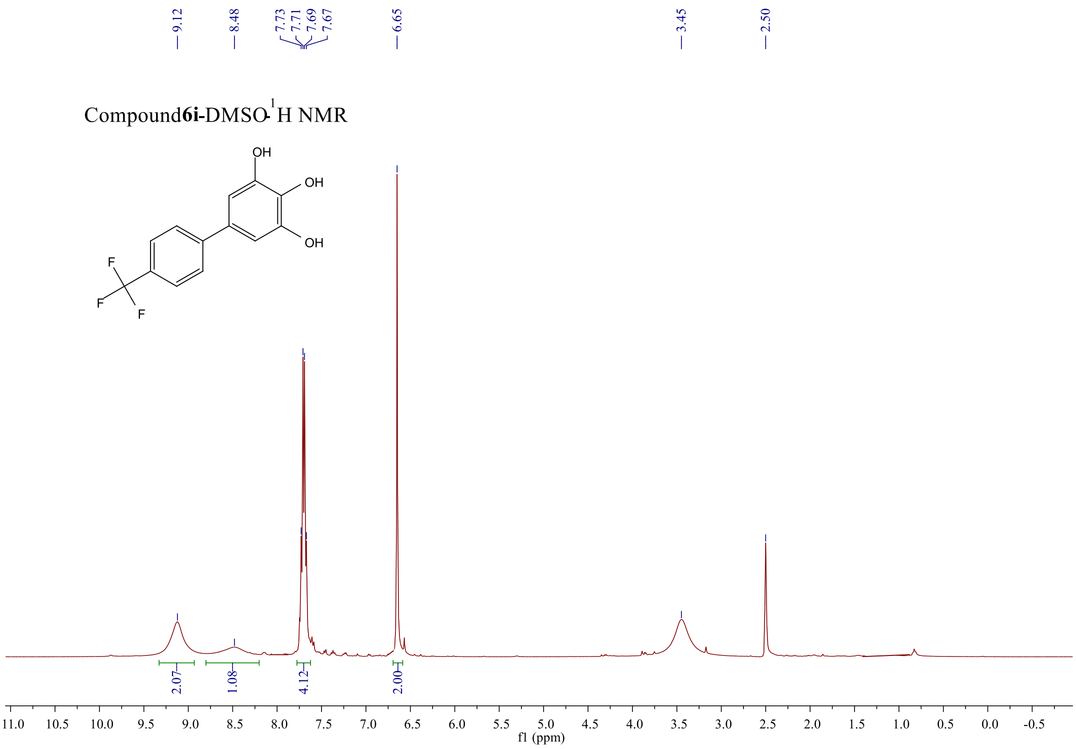

^{1}H NMR spectrum of compound **6i** (20 mg in 0.6 mL DMSO-d_6, 25 °C, Scan times ns = 32)

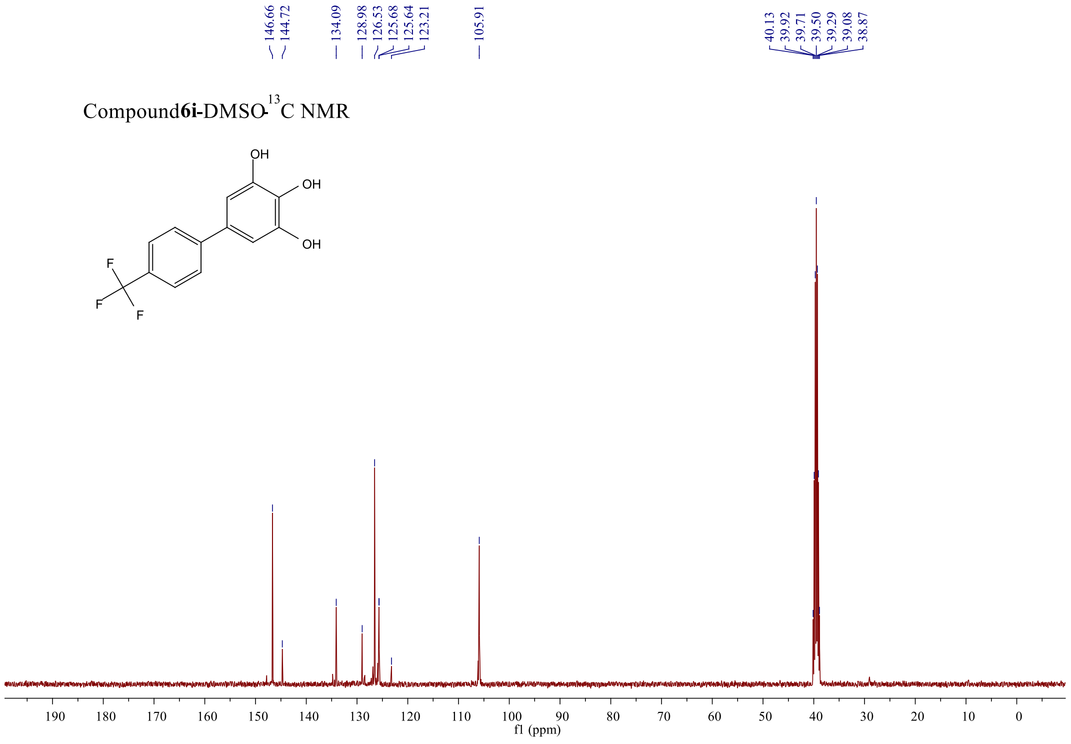

^{13}C NMR spectrum of compound **6i** (35 mg in 0.6 mL DMSO-d_6, 25 °C, Scan times ns = 1024)

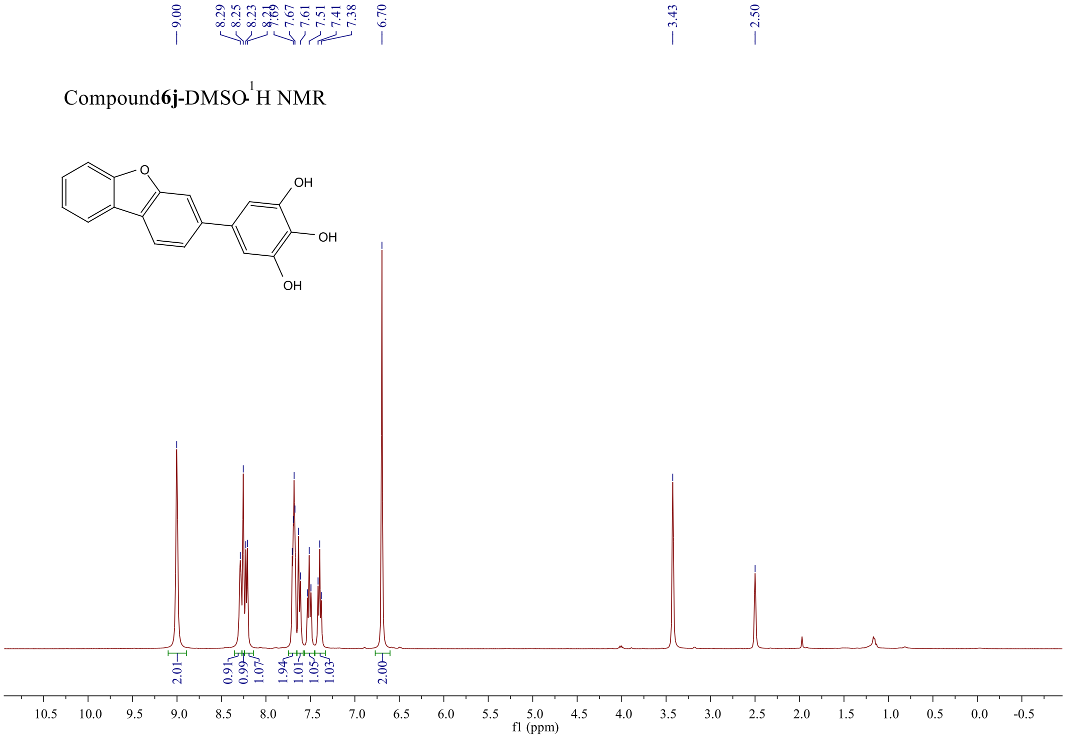

^{1}H NMR spectrum of compound **6j** (20 mg in 0.6 mL DMSO-d_6, 25 °C, Scan times ns = 32)

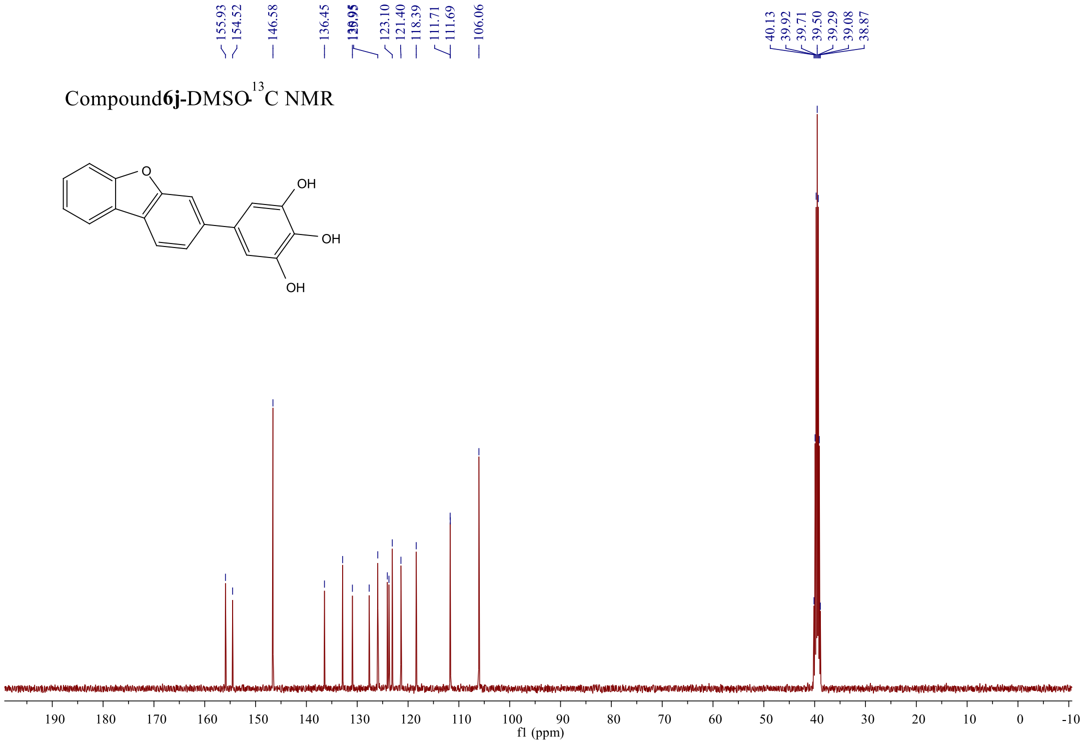

^{13}C NMR spectrum of compound **6j** (35 mg in 0.6 mL DMSO-d_6, 25 °C, Scan times ns = 1024)

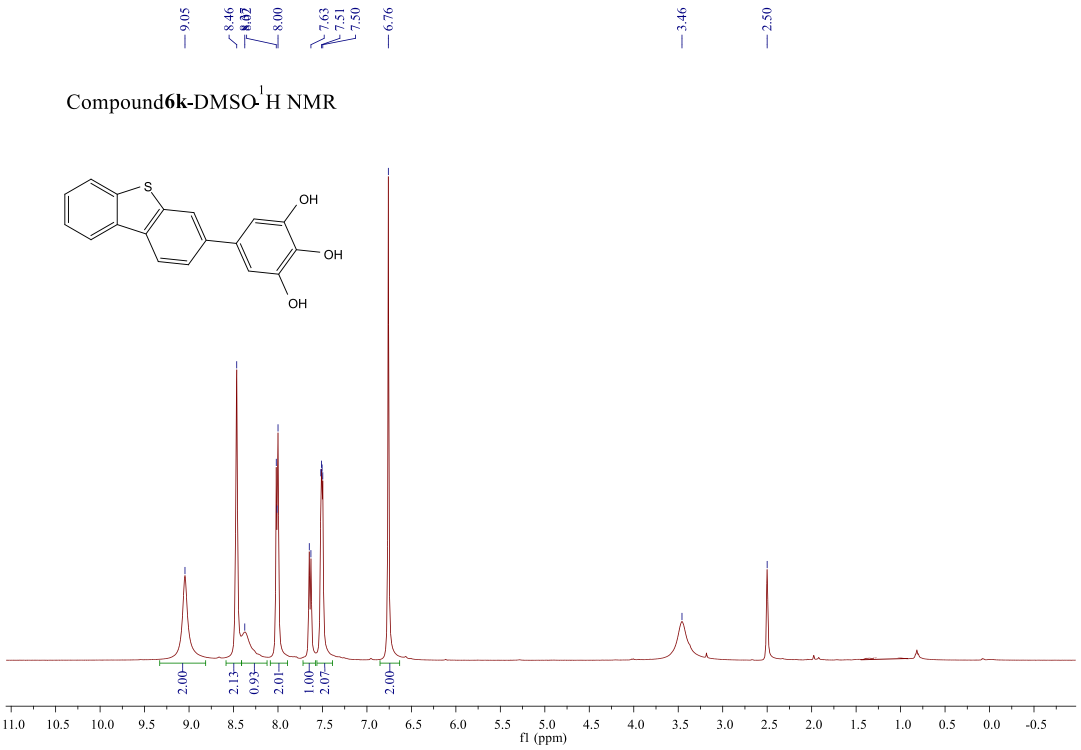

^{1}H NMR spectrum of compound **6k** (20 mg in 0.6 mL DMSO-d_6, 25 °C, Scan times ns = 32)

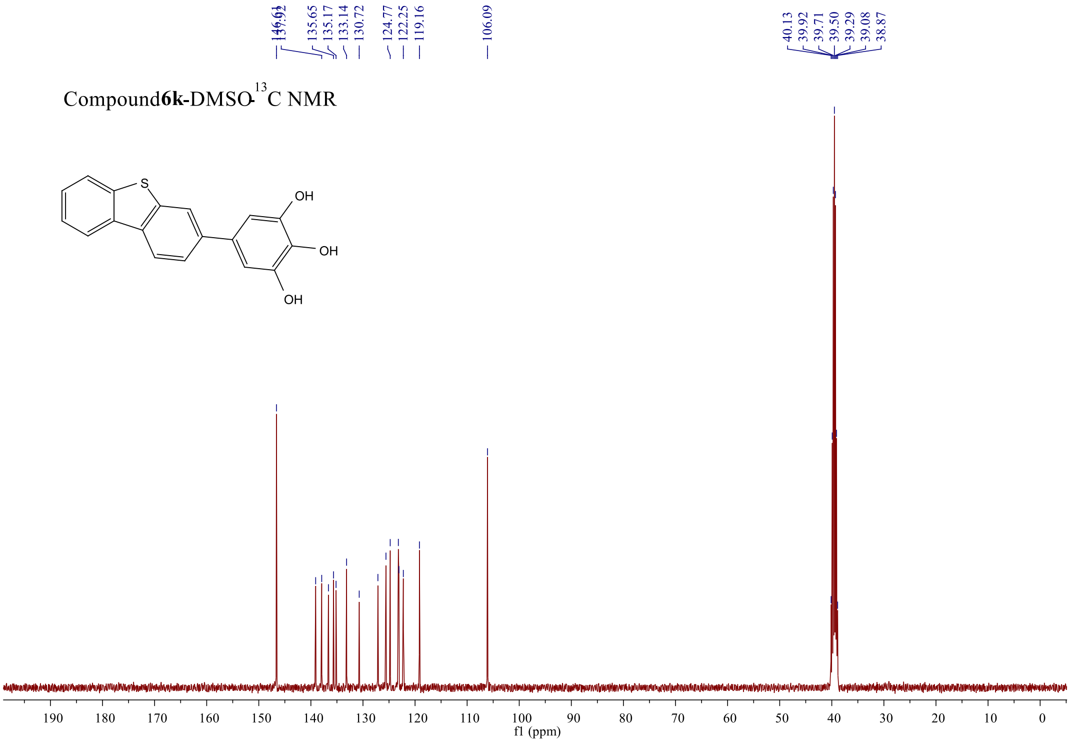

^{13}C NMR spectrum of compound **6k** (35 mg in 0.6 mL DMSO-d_6, 25 °C, Scan times ns = 1024)

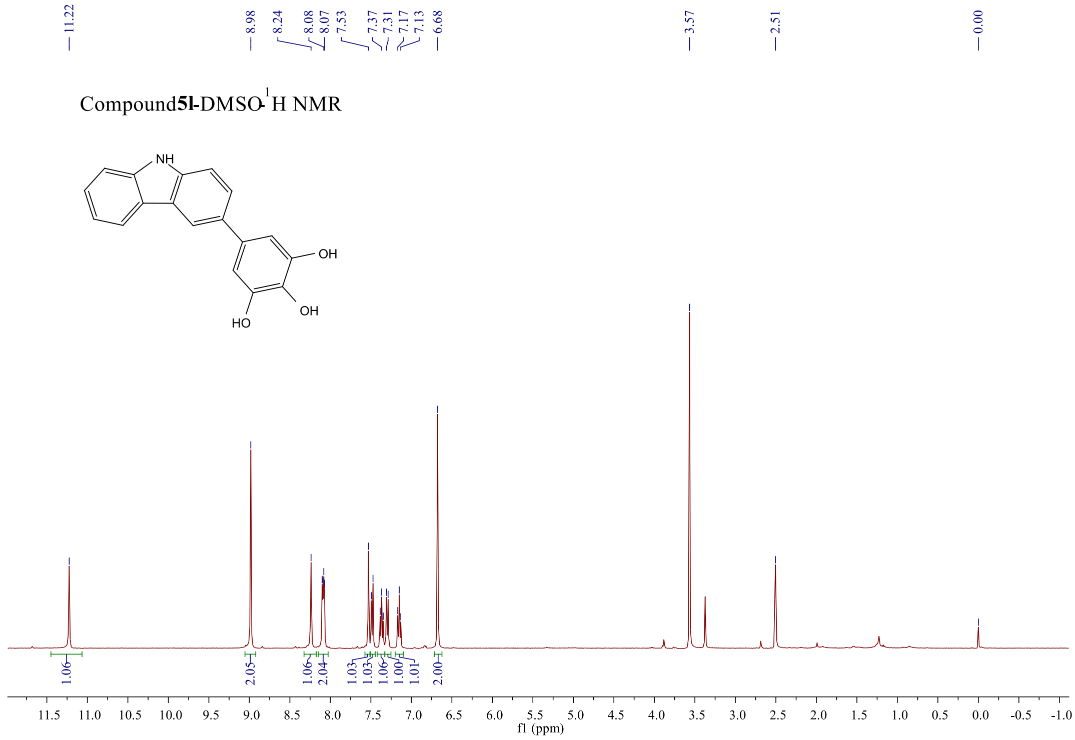

^{1}H NMR spectrum of compound **6l** (20 mg in 0.6 mL DMSO-d_6, 25 °C, Scan times ns = 32)

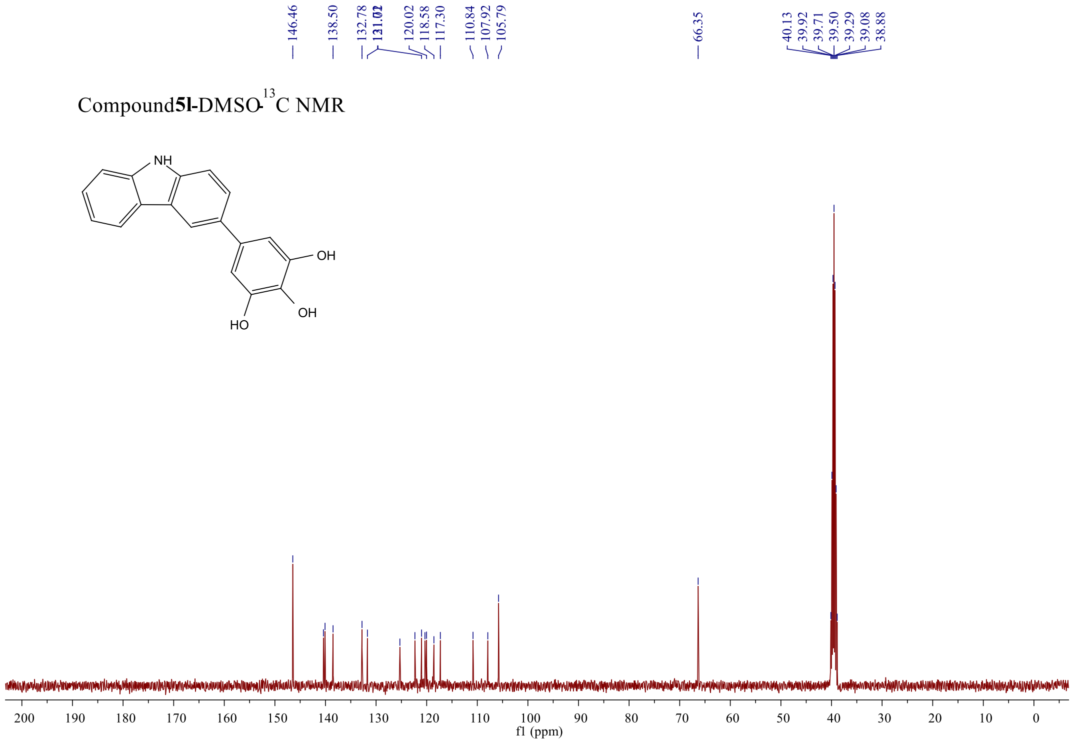

^{13}C NMR spectrum of compound **6l** (35 mg in 0.6 mL DMSO-d_6, 25 °C, Scan times ns = 1024)

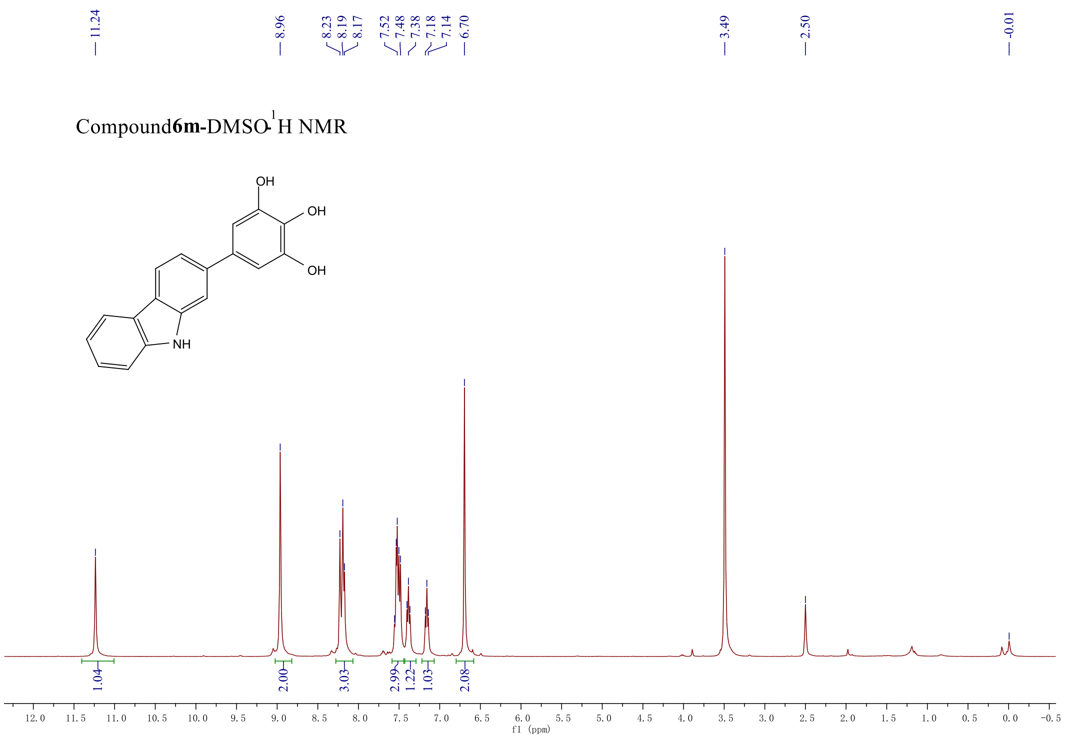

^{1}H NMR spectrum of compound **6m** (20 mg in 0.6 mL DMSO-d_6, 25 °C, Scan times ns = 32)

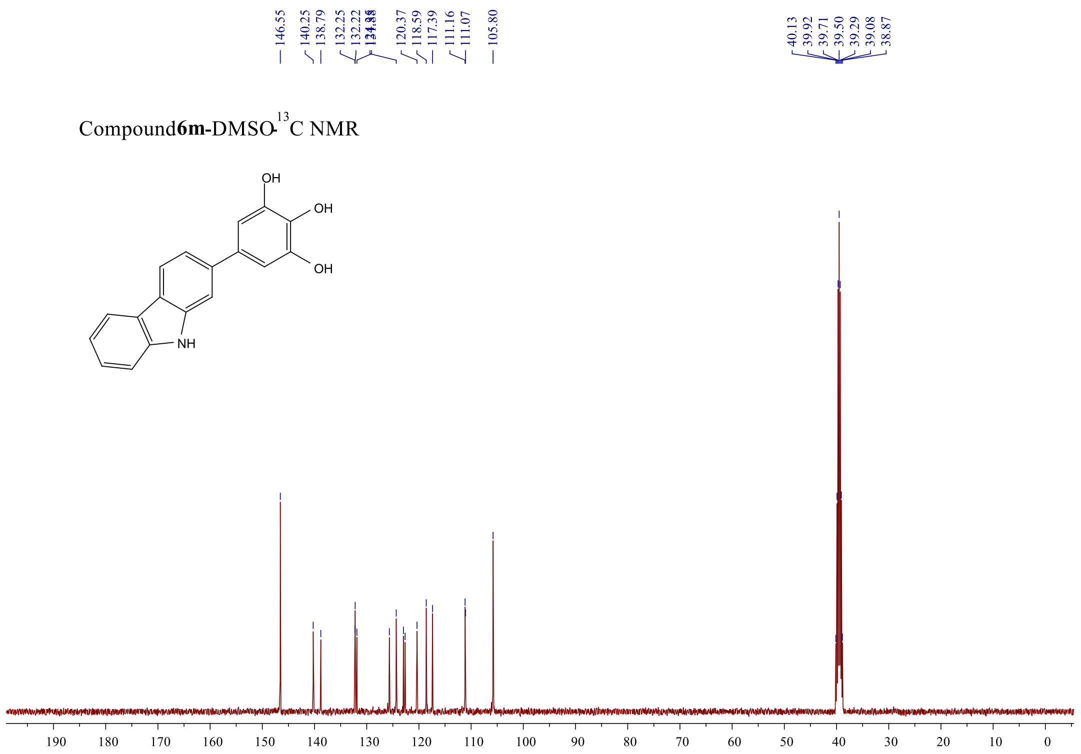

^{13}C NMR spectrum of compound **6m** (35 mg in 0.6 mL DMSO-d_6, 25 °C, Scan times ns = 1024)

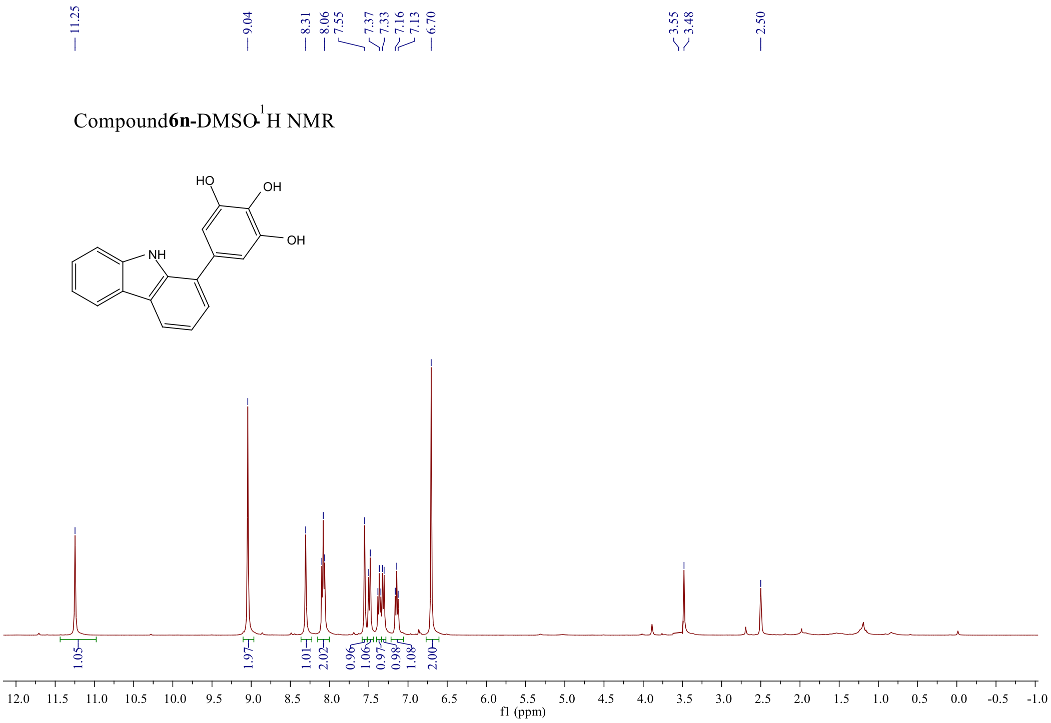

^{1}H NMR spectrum of compound **6n** (20 mg in 0.6 mL DMSO-d_6, 25 °C, Scan times ns = 32)

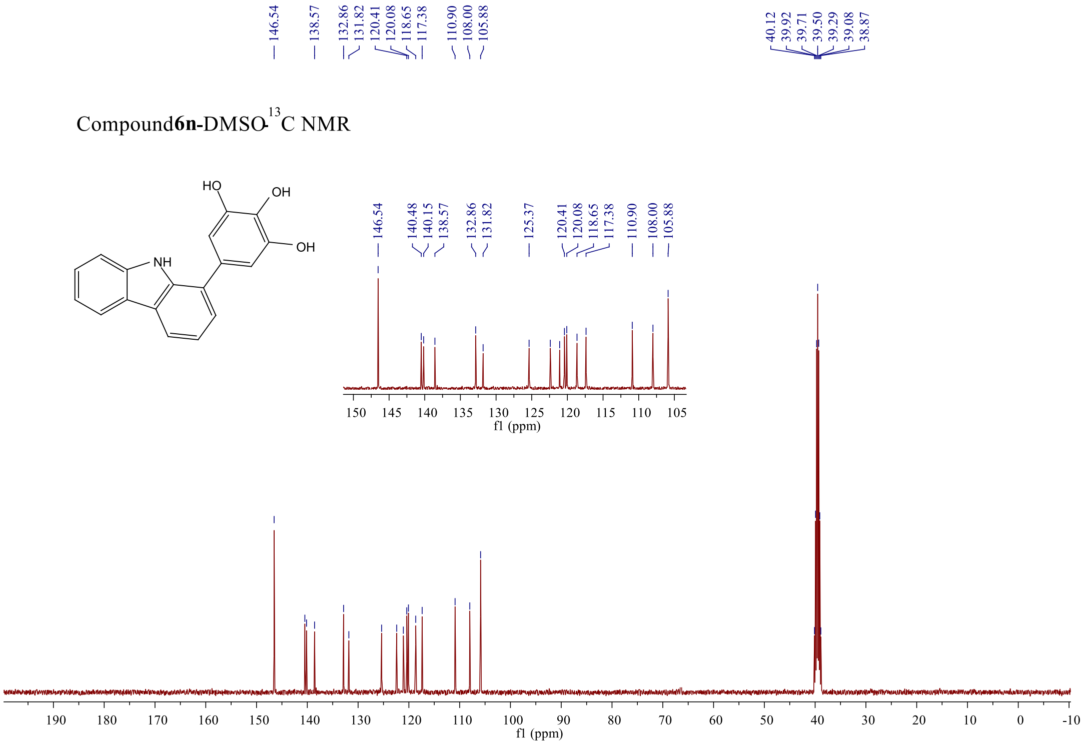

^{13}C NMR spectrum of compound **6n** (35 mg in 0.6 mL DMSO-d_6, 25 °C, Scan times ns = 1024)
